# Hijacking the Peptidoglycan Recycling Pathway of *Escherichia coli* to Produce Muropeptides

**DOI:** 10.1002/chem.202202991

**Published:** 2022-12-05

**Authors:** Antoine Rousseau, Julie Michaud, Stéphanie Pradeau, Sylvie Armand, Sylvain Cottaz, Emeline Richard, Sébastien Fort

**Affiliations:** ^1^ Univ. Grenoble Alpes, CNRS, CERMAV 38000 Grenoble France

**Keywords:** diaminopimelic acid, *Escherichia coli*, metabolic engineering, muropeptides, peptidoglycan

## Abstract

Soluble fragments of peptidoglycan called muropeptides are released from the cell wall of bacteria as part of their metabolism or as a result of biological stresses. These compounds trigger immune responses in mammals and plants. In bacteria, they play a major role in the induction of antibiotic resistance. The development of efficient methods to produce muropeptides is, therefore, desirable both to address their mechanism of action and to design new antibacterial and immunostimulant agents. Herein, we engineered the peptidoglycan recycling pathway of *Escherichia coli* to produce *N*‐acetyl‐β‐D‐glucosaminyl‐(1→4)‐1,6‐anhydro‐*N*‐acetyl‐β‐D‐muramic acid (GlcNAc‐anhMurNAc), a common precursor of Gram‐negative and Gram‐positive muropeptides. Inactivation of the hexosaminidase *nagZ* gene allowed the efficient production of this key disaccharide, providing access to Gram‐positive muropeptides through subsequent chemical peptide conjugation. *E. coli* strains deficient in both NagZ hexosaminidase and amidase activities further enabled the in vivo production of Gram‐negative muropeptides containing *meso*‐diaminopimelic acid, a rarely available amino acid.

## Graphical abstract

**A straightforward access** to Gram‐negative and Gram‐positive muropeptides was developed by a chemo‐enzymatic strategy based on metabolically engineered *E.coli* cells.

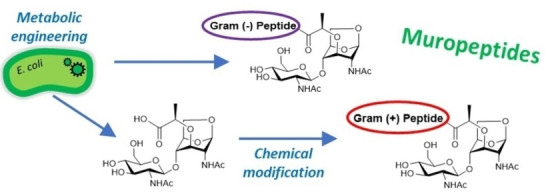

## Introduction

**Figure 1:**
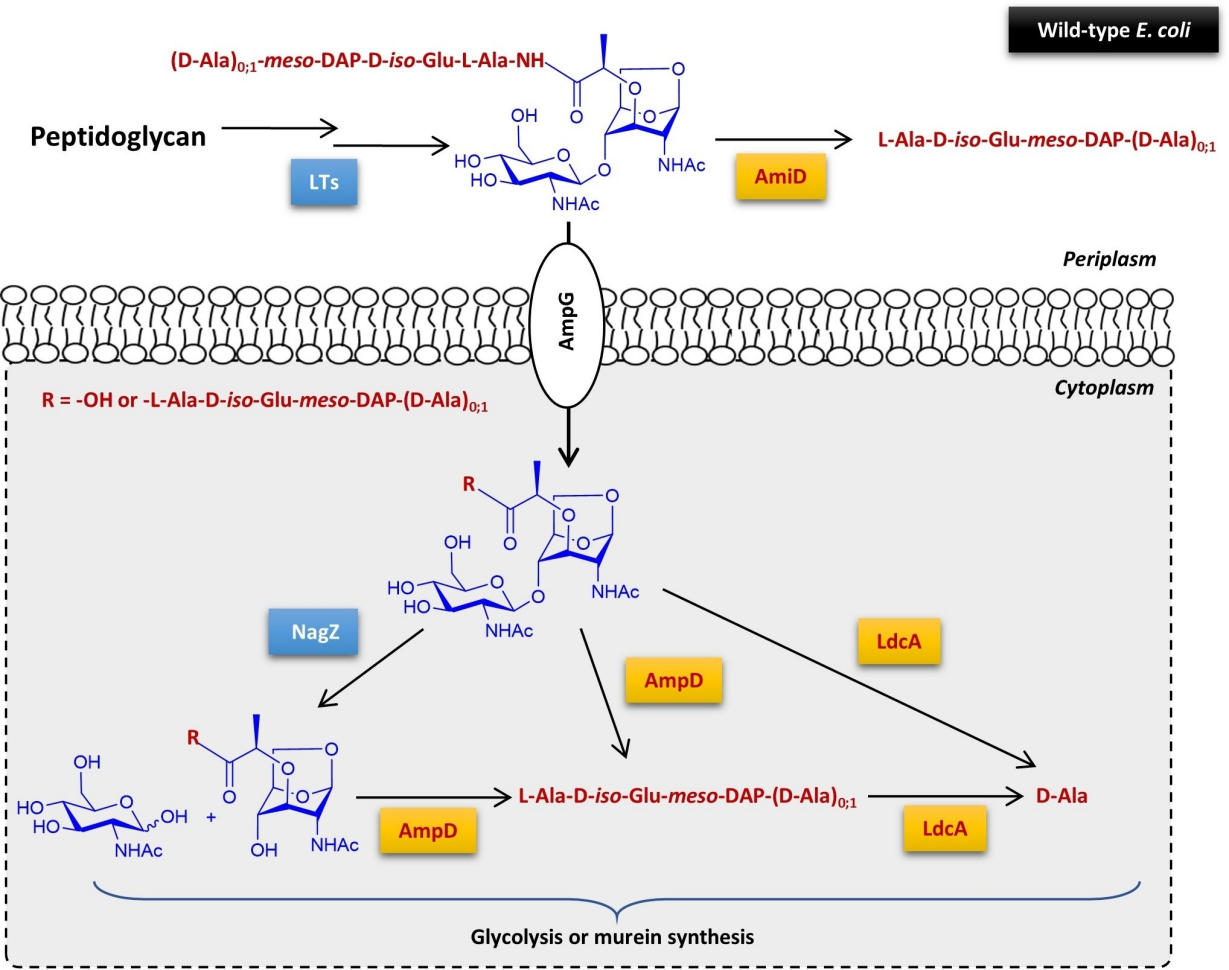
*E. coli* peptidoglycan recycling pathway. (LTs: lytic transglycosylases; AmiD and AmpD: *N*‐acetylmuramoyl‐L‐alanine amidases; AmpG: permease; LdcA: L,D‐carboxypeptidase; NagZ: *N*‐acetyl‐β‐D‐hexosaminidase)

Peptidoglycan (PG), or murein, is a major component of the bacterial cell wall in both Gram‐positive and Gram‐negative species.^[1]^ This heteropolymer consists of glycan strands that are crosslinked by peptides. The saccharide backbone is composed of alternating units of *N*‐acetylglucosamine (GlcNAc) and *N*‐acetylmuramic acid (MurNAc) linked by β‐1,4‐glycosidic bonds. The peptide stems are connected to the glycan chains via the lactyl groups of the MurNAc residues and their composition, which consists of three to five alternating L‐ and D‐amino acids, varies with the bacterial species. During their life cycle, bacteria recycle a large part of their PG.^[2]^ In *E. coli*, a series of enzymes degrade almost 50 % of the murein every generation (Figure 1). The tetrapeptide disaccharide GlcNAc‐anhMurNAc‐L‐Ala‐D‐*iso*‐Glu‐*meso*‐DAP‐D‐Ala (*meso*‐DAP stands for *meso*‐diaminopimelic acid), also referred to as tracheal cytotoxin (TCT) due to its toxicity to tracheal epithelial cells, is the main intermediate of PG degradation by lytic transglycosylases (LTs). LTs also produce the tripeptide disaccharide GlcNAc‐anhMurNAc‐L‐Ala‐D‐*iso*‐Glu‐*meso*‐DAP, but to a lesser extent. Both products are partly modified by the periplasmic amidase AmiD, which removes the peptide stem, or are transported into the cytoplasm by the permease AmpG. In the cytoplasm, the tri‐ and tetrapeptide GlcNAc‐anhMurNAc as well as the GlcNAc‐anhMurNAc, which is also internalized by AmpG, are processed by three key enzymes: *N*‐acetyl‐β‐D‐hexosaminidase NagZ hydrolyzes the glycosidic bond between the GlcNAc and anhMurNAc units, the endopeptidase AmpD releases the peptide stem from anhMurNAc and L,D‐carboxypeptidase LdcA removes the terminal D‐Ala amino acid from the tetrapeptide.[^3^, ^4^] These enzymes act mainly in a sequential manner. Nevertheless, LdcA^[5]^ and AmiD^[6]^ also display noticeable activity on the tri‐and tetrapeptide GlcNAc‐anhMurNAc. The resulting elementary blocks are then degraded through glycolysis or recycled for murein biosynthesis.

**Figure 2:**
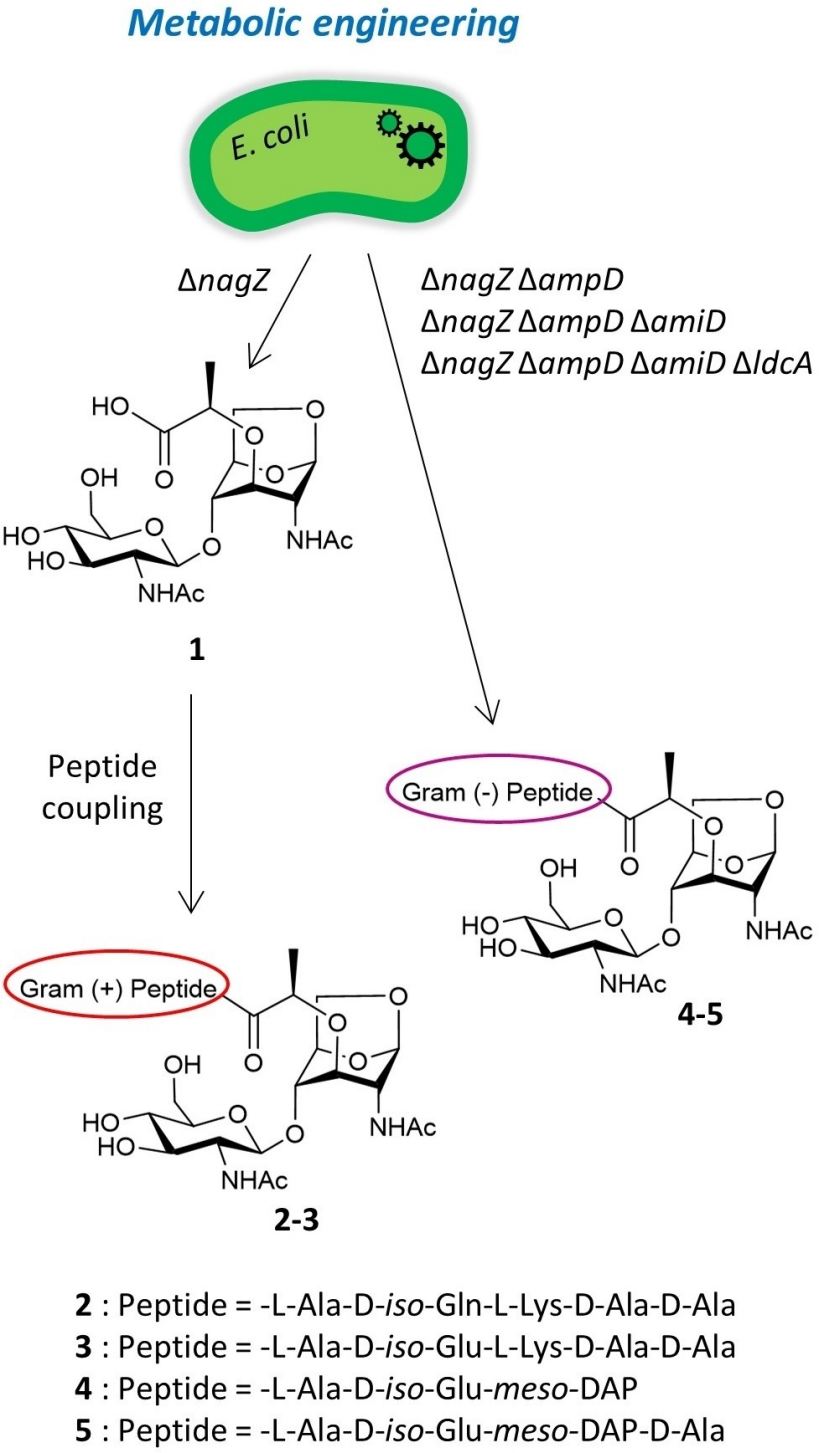
Synthesis strategy for Gram‐negative and Gram‐positive GlcNAc‐anhMurNAc‐based muropeptides using metabolically engineered *E. coli* strains.

A minor fraction (about 7 %) of the soluble PG fragments, also called muropeptides, are not recycled, but released into the immediate surroundings of the bacteria where they are perceived as pathogen‐associated molecular patterns (PAMPs) by other species.^[7]^ These muropeptides trigger innate immune responses in animals, plants and some insects.^[8]^ TCT has been identified as a virulence factor of *Bordetella pertussis* ^[9]^ and *Neisseria gonorrhoeae*,^[10]^ the causative agents of whooping cough and gonococcal infection, respectively. In both diseases, colonization of the host is associated with the secretion of the tetrapeptide disaccharide, which causes inflammation and cell death. In the Gram‐negative bacterium *Pseudomonas aeruginosa*, the pentapeptide disaccharide GlcNAc‐anhMurNAc‐L‐Ala‐D‐*iso*‐Glu‐*meso*‐DAP‐D‐Ala‐D‐Ala can participate in β‐lactam antibiotic resistance mechanisms.^[11]^ The peptide moiety of muropeptides plays a critical role in their activity and host specificity. The tripeptide disaccharide GlcNAc‐anhMurNAc‐L‐Ala‐D‐*iso*‐Glu‐*meso*‐DAP triggers the activation of innate immunity in humans by interacting with the nucleotide‐binding oligomerization domain 1 (NOD1),^[12]^ whereas TCT, the same disaccharide bound to the tetrapeptide L‐Ala‐D‐*iso*‐Glu‐*meso*‐DAP‐D‐Ala is specific to murine NOD1.^[13]^ In addition, TCT triggers innate immunity in *Drosophila* by activating the peptidoglycan recognition protein LC (PGRP‐LC), but the bare disaccharide does not.^[14]^ The muramyl dipeptide MurNAc‐L‐Ala‐D‐*iso*‐Gln (MDP), produced by Gram‐negative species and the Gram‐positive *Streptococcus pneumonia*, is an active component of the complete Freund's adjuvant, the most widely used and effective adjuvant for experimental antibody production. MDP binds to the mammalian innate immune receptor NOD2, inducing the production of pro‐inflammatory cytokines.^[15]^ A recent study in mice showed that MDP produced by intestinal bacteria is also perceived by hypothalamic neurons to regulate feeding behavior and body temperature.^[16]^ In this manner, bacterial microbiota may be used by the brain as an indirect measure of food intake or as a direct measure of bacterial expansion or death attributable to food intake. The clinical uses and therapeutic potential of muropeptides have stimulated interest in the synthesis of this class of molecules. Since the seminal works of Sinaÿ et al. in 1973^[17]^ and Paulsen et al. in 1986^[18]^, most of the syntheses of PG oligomers and muropeptides reported in the literature relied on a chemical strategy.^[19]^ Despite its versatility and high yields, this approach requires a huge number of steps.[^20^, ^21^, ^22^] In 2000, in the course of their research on NagZ, Park et al. reported that the enzyme is not essential to the bacterium and that a *nagZ*‐deficient *E. coli* mutant accumulates GlcNAc‐anhMurNAc in its cytoplasm.^[23]^ However, these findings were exclusively based on chromatographic analytical data using radiolabeled muropeptides, and the products were not isolated on a preparative scale nor characterized. Here, we designed four *E. coli* strains using metabolic engineering and by cultivating them at high cell density we produced Gram‐negative and Gram‐positive muropeptides on a preparative scale. The *E. coli* Δ*nagZ* strain was prepared to access the disaccharide GlcNAc‐anhMurNAc as a precursor of Gram‐positive muropeptides through chemical conjugation with synthetic peptides. Strains with additional knockouts of the *ampD*, *amiD* and *ldcA* genes were designed to produce Gram‐negative muropeptides containing a *meso*‐DAP amino acid, whose chemical synthesis is extremely complex (Figure 2).

## Results and Discussion

### Production of the disaccharide GlcNAc‐1,6‐anhMurNAc (1)

**Figure 3:**
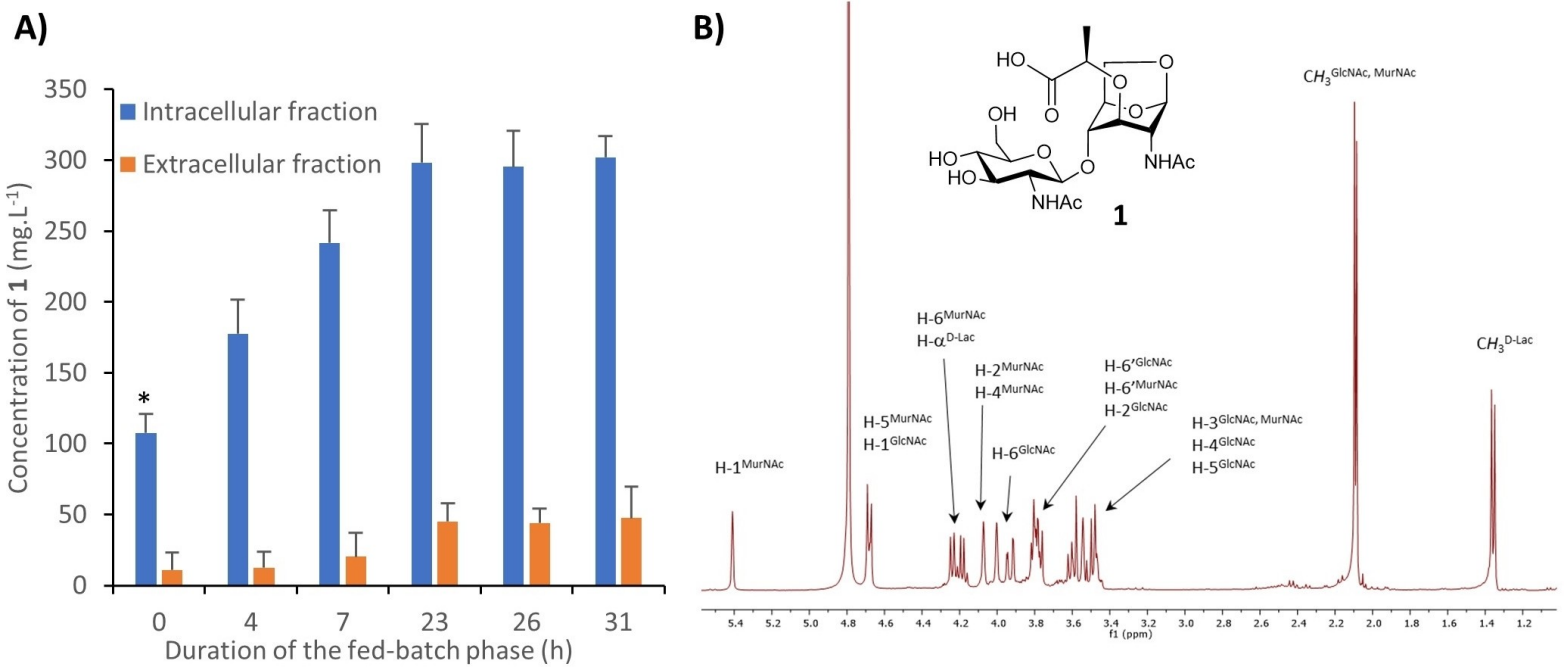
A) Quantification of GlcNAc‐1,6‐anhMurNAc **1** in the intracellular and extracellular fractions, as determined using HPAEC‐PAD at different time points during the fed‐batch phase (* **1** starts to accumulate in the intracellular fraction during the batch phase (≈16 h)). B) ^1^H NMR spectrum of **1** in D_2_O at 298 K.

To produce the GlcNAc‐anhMurNAc disaccharide **1**, the *nagZ* gene of the *E. coli* K12 strain DH1 (DSM 4235) was inactivated. First, bacteria were grown in minimal medium to confirm the viability of the *E. coli* Δ*nagZ* strain under high cell density culture conditions and to validate the accumulation of **1**. The impairment of the PG recycling pathway was not lethal to the bacterium and the presence of the disaccharide in the cytoplasm was confirmed by mass spectrometry analysis (data not shown). As a control, the wild type strain was grown under the same conditions and the disaccharide **1** was not detected. High cell density culture of metabolically engineered bacteria in a bioreactor is an effective approach to produce large amounts of oligo‐ and polysaccharides.[^24^, ^25^, ^26^, ^27^] This process takes place in two phases: an initial exponential growth phase, which starts at the inoculation of the bioreactor and lasts until exhaustion of the carbon sources initially added to the medium, then a fed‐batch phase with a high glycerol feeding rate. The production of **1** was carried out in 3 L bioreactors containing 1.5 L of medium and monitored using high‐performance anion‐exchange chromatography with pulsed amperometric detection (HPAEC‐PAD). The accumulation of **1** in the intracellular fraction began during the exponential growth phase, at the end of which its concentration reached 100 mg L^−1^. The production of **1** increased to approximately 300 mg L^−1^ then remained constant after 23 h of the fed‐batch phase, although the number of cells was in constant progression during the culture (Supporting Information Figure S1). Inversely, the disaccharide concentration in the extracellular fraction was very low until 23 h of the fed‐batch phase, when it reached 50 mg L^−1^ (Figure 3A). The late accumulation of **1** in the extracellular medium was likely due to product diffusion or cell lysis. Attempts to increase the overall production of the disaccharide by modification of the culture medium and the culture conditions were unsuccessful. A proposed explanation for this result is that production of **1** by *E. coli* Δ*nagZ* occurs at the beginning of the cell division and then slows down, probably due to PG homeostasis.[^29^, ^30^] Because of *nagZ* inactivation, the supply of UDP‐MurNAc‐pentapeptide is impaired, which may negatively regulate the de novo PG biosynthesis and restrict the accumulation of **1**.^[30]^ Considering the limited amount of **1** in the extracellular fraction with regard to its overall production and the effort to isolate a negatively charged disaccharide from a culture medium rich in ionic species, the culture was stopped 23 h after the beginning of the fed‐batch phase and the disaccharide was purified from the intracellular compartment exclusively. After cell lysis by heat shock, **1** was isolated by adsorption on activated charcoal and then purified successively using silica gel flash chromatography and size‐exclusion chromatography (SEC). The structure of **1**, characterized using ^1^H and ^13^C NMR (Figure 3B and Supporting Information S2) was in perfect agreement with that reported for the same compound prepared by chemical synthesis.^[28]^ The average production yield after purification by chromatography was 200 mg L^−1^. This result, although slightly lower than the yield estimated by ion‐exchange chromatography, was highly satisfactory considering the difficulty in purifying this class of compound from a bacterial medium. Finally, the engineered *E. coli* Δ*nagZ* strain enabled the production of 300 mg of GlcNAc‐anhMurNAc from a 1.5 L culture over two days, which makes this process very efficient compared with chemical synthesis. The method has proved reproducible and a culture in a 7 L reactor (4 liters of culture medium) afforded the expected 800 mg of product.

### Production of Gram‐positive disaccharide muropeptides

**Scheme 1:**
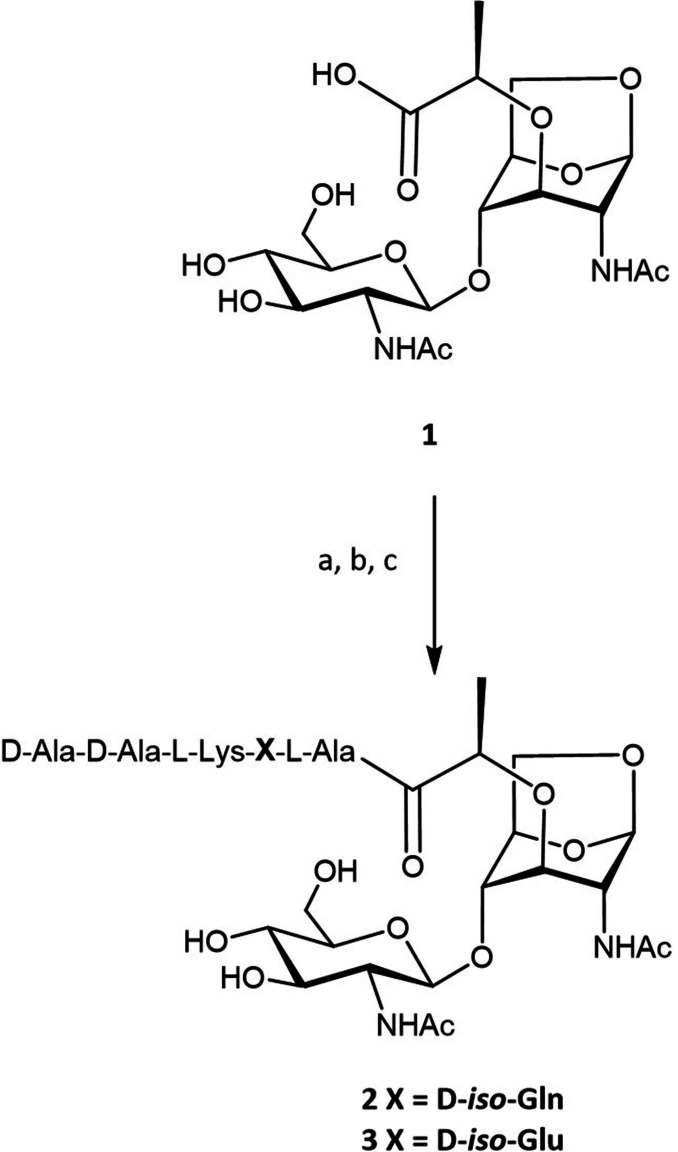
Synthesis of Gram‐positive muropeptides. a) HOAt, EDC, DIPEA, pentapeptide TFA salt, DMF; b) 20 % piperidine in DMF; c) 0.1 M ammonium carbonate, 31 % and 42 % over three steps for **2** and **3**, respectively.

With a significant amount of GlcNAc‐anhMurNAc available, the preparation of muropeptides was undertaken. Most Gram‐positive bacteria, including the highly pathogenic *Staphylococcus aureus*, *Streptococcus pneumoniae or Enterococcus faecalis* multidrug‐resistant strains, display the peptide stem L‐Ala‐D‐*iso*‐Gln‐L–Lys‐D‐Ala‐D‐Ala.^[31]^ The protected pentapeptide L‐Ala‐D‐*iso*‐Gln‐L–Lys(Fmoc)‐D‐Ala‐D‐Ala‐OMe was thus prepared using solution‐phase synthesis (Supporting Information S3) and conjugated to **1** using HOAt/EDC/DIPEA coupling conditions. After deprotection of the Fmoc using piperidine and saponification of the methyl ester in 0.1 M ammonium carbonate, the pentapeptide disaccharide **2** (Scheme 1) was isolated with a yield of 31 % over the three steps and fully characterized by ^1^H and ^13^C NMR (Supporting Information S4). During PG synthesis in Gram‐positive bacteria, the peptide stem of the lipid II precursor is modified on the second amino acid. The D‐glutamic residue is converted into a D‐*iso*‐glutamine by the MurT/GatD enzyme complex.^[32]^ MurT/GatD, whose depletion causes susceptibility to β‐lactams in *S. aureus* methicillin‐resistant strains, is a target for the development of new antibacterial treatments. Considering that **1** could be a valuable precursor of lipid II to study the MurT/GatD enzyme complex, we conjugated the pentapeptide L‐Ala‐D‐*iso*‐Glu(OMe)‐L–Lys(Fmoc)‐D‐Ala‐D‐Ala(OMe)^[33]^ to GlcNAc‐anhMurNAc. Coupling **1** with the protected peptide followed by deprotection afforded **3** with a yield of 42 %. Characterization by ^1^H and ^13^C NMR (Supporting Information S5) was in perfect agreement with the data reported for the same compound produced by total chemical synthesis.^[20]^

### Production of Gram‐negative disaccharide muropeptides

The Gram‐negative bacterial PG is characterized by the presence of the non‐canonical amino acid *meso*‐DAP residue at the third position of the peptide stem. The synthesis of this peptide is a daunting task because an orthogonally protected *meso*‐DAP must be used. Unfortunately, this building block is not commercially available and its preparation by chemical[^34^, ^35^, ^36^, ^37^] or chemo‐enzymatic^[38]^ approaches requires a dozen steps. *E. coli*, which is a Gram‐negative bacterium, synthesizes and incorporates *meso*‐DAP in its PG. With the aim of producing *meso*‐DAP‐containing muropeptide disaccharides, we developed *E. coli* strains deficient in amidase and L,D‐carboxypeptidase activities starting from the Δ*nag*Z mutant.

Based on an HPLC analysis, Cheng et al. previously reported that *E. coli TP78* Δ*nagZ* Δ*ampD* accumulates a larger amount of tripeptide disaccharide **4** than **1** in its cytoplasm.^[23]^ This result was later supported by other data showing that AmpD is not only active on peptide anhMurNAc, but also acts on peptide GlcNAc‐anhMurNAc.^[6]^ The *E. coli* Δ*nagZ* Δ*ampD* strain was thus designed to produce the tripeptide disaccharide **4**. High cell density culture of this strain and purification of the bacterial cytoplasmic fraction by charcoal adsorption followed by SEC afforded the disaccharide **1** at 190 mg L^−1^, whereas the tripeptide disaccharide **4**, whose chemical structure has been unambiguously confirmed by NMR and mass spectrometry (Supporting Information S6), was only produced at 32 mg L^−1^ (Table 1). This result contrasts with that reported previously for **1**.^[23]^

**Table 1:** Production yield (in mg L^−1^ culture medium) after chromatographic purification of Gram‐negative peptidoglycan disaccharides produced by high cell density culture of metabolically engineered *E. coli* strains. nd=not detected.

	*E. coli* strains			
	Δ*nagZ*	Δ*nagZ*	Δ*nagZ*	Δ*nagZ*
		Δ*ampD*	Δ*ampD*	Δ*ampD*
			Δ*amiD*	Δ*amiD*
				Δ*ldcA*
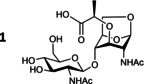	200	190	52	28
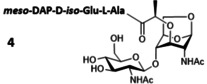	nd	32	305	90
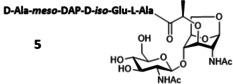	nd	nd	nd	360

Because the transport of the tripeptide disaccharide **4** to the cytoplasm by AmpG was previously reported to be of the same order of magnitude as that of disaccharide **1**,^[39]^ we assumed that the low amount of **4** isolated from the Δ*nagZ* Δ*ampD* strain was due to a partial degradation by AmiD in the periplasm. The strain *E. coli* Δ*nagZ* Δ*ampD* Δ*amiD* was then designed to increase the production of **4**. Under similar fermentation and purification conditions, the production of the tripeptide disaccharide **4** increased to 305 mg L^−1^ (Table 1) and that of **1** dropped to 52 mg L^−1^. Suppression of AmiD led to a significant improvement in the production of **4**. The presence of other periplasmic amidases such as AmiA, B or C^[40]^ likely caused the formation of **1**, but in a relatively low amount. Considering that a knockout of these amidases would not significantly improve the production of **4**, we then focused on abolishing the activity of LdcA to produce the tetrapeptide GlcNAc‐anhMurNAc **5**. In effect, although LdcA shows its highest activity on the tetrapeptide L‐Ala‐D‐*iso*‐Glu‐*meso*‐DAP‐D‐Ala, this enzyme also hydrolyzes the terminal alanine residue on **5**.^[5]^ High cell density culture of the *E. coli* Δ*nagZ* Δ*ampD* Δ*amiD* Δ*ldcA* strain produced a mixture of **1**, **4** and **5** (Table 1). As expected, disaccharide **1** was isolated with a low yield of 28 mg L^−1^ after purification by SEC. The tri‐ and tetrapeptide derivatives **4** and **5** were recovered with a yield of 450 mg L^−1^ culture medium as a 20 : 80 w/w mixture. This ratio was determined by ^1^H NMR and analytical reversed‐phase HPLC. We were able to isolate a pure sample of **5** using SEC to be fully characterized by ^1^H and ^13^C NMR (Supporting Information S7). However, the low difference in molecular weight between **4** and **5** did not allow a total separation of the two products by this method. Instead, reversed‐phase HPLC should be used, as suggested by the efficient separation of compounds on an analytical scale (Supporting Information S8).

## Conclusion

To conclude, metabolic engineering of the PG recycling pathway of *E. coli* provided straightforward access to disaccharide muropeptides on a preparative scale. High cell density culture of a *nagZ‐*deficient strain produced GlcNAc‐anhMurNAc at 200 mg L^−1^. This disaccharide was further efficiently converted to Gram‐positive muropeptides. *E. coli* strains lacking the hexosaminidase NagZ as well as amidases (AmiD, AmpD and LdcA) led to the production of tri‐ and tetrapeptide GlcNAc‐anhMurNAc containing a *meso*‐DAP group with similar efficiency. These muropeptides open new perspectives for the controlled enzymatic synthesis of PG fragments. We recently reported a lysozyme‐derived glycosynthase for this purpose.^[41]^ The building block reported in the present work will be converted into glycosyl donors for enzymatic elongation by acetolysis of the 1,6‐anhMurNAc and activation of the anomeric position. In addition, the Gram‐negative muropeptides produced in vivo may serve as a source of *meso*‐DAP‐containing tri‐ and tetrapeptides whose chemical syntheses are extremely challenging. Our findings show that *meso*‐DAP‐containing tri‐ and tetrapeptides can potentially be synthesized in vitro using a recombinant amidase AmiD. Finally, the methodology reported here provides access to useful molecular probes for studying bacterial cell wall metabolim and antimicrobial resistance mechanisms.

## Experimental Section

**General**: Reagents for bacterial media were obtained from Euromedex (Mundolsheim, France) and Invitrogen (Cergy‐Pontoise, France). Molecular biology reagents were obtained from Invitrogen, Euromedex, Macherey‐Nagel (Hoerd, France) and Thermo Fisher Scientific (Villebon‐sur‐Yvette, France). The PrimeSTAR GLX premix and the ligation mix were from Takara (Saint‐Germain‐en‐Laye, France). Oligonucleotides were purchased from Eurofins MWG Operon (city, Germany). Chemicals were of reagent grade, purchased from Sigma‐Aldrich (Saint‐Quentin‐Fallavier, France) or TCI (Europe) and used directly without further purification.

NMR spectra were recorded at 298 K in D_2_O with a Bruker Avance III 400 MHz spectrometer (Bruker, Wissembourg, France). For ^1^H NMR, the solvent residual peak was used as an internal standard at 4.79 ppm. Data for ^1^H NMR are reported in the conventional form: chemical shift (*δ* ppm) (multiplicity (s=singlet, d=doublet, t=triplet, q=quartet, m=multiplet), coupling constant (Hz), integration). Compounds **1**–**5** were completely assigned using a combination of COSY, HMQC, and HMBC experiments.

Reactions were monitored using thin layer chromatography (TLC) on silica gel (Merck Silica gel 60 F254, Darmstadt, Germany). Carbohydrates or peptides were visualized with either a 30 % ammonium bisulfate solution in H_2_O or diphenylamine‐aniline‐phosphoric acid (4 g diphenylamine, 4 mL aniline, 2 mL 37 % HCl, 20 mL 85 % H_3_PO_4_ in 200 mL ethyl acetate) staining solution followed by heating with a heat gun.

The intra‐ and extracellular concentrations of GlcNAc‐anhMurNAc **1** were determined against a calibration curve obtained with pure disaccharide using high‐performance anion‐exchange chromatography with pulsed amperometric detection (HPAEC‐PAD, ICS‐6000 Dionex) equipped with a CarboPac PA1 guard column and CarboPac PA1 analytical column (Thermo Fisher Scientific) thermostated at 30 °C. Samples were filtered on 0.22 μM filters prior to injection. Disaccharide **1** was eluted with a 0 % to 50 % 1 M sodium acetate gradient in 0.1 M sodium hydroxide for 30 minutes at 1 mL.min^−1^.

Compounds were purified using flash‐column chromatography (wet‐packed silica, Merck 0.04–0.063 mm), adsorption on activated charcoal and preparative size‐exclusion chromatography (SEC) or high‐performance liquid chromatography (HPLC). SEC was performed using three Hiload Superdex 30 columns (10‐300GL) in series with a refractive index detector RI Iota 2 (Interchim, Montluçon, France). Elution was carried out with 0.1 M ammonium carbonate at a flow rate of 1.2 mL.min^−1^. HPLC was conducted on an Ultimate 3000 (Thermo Fisher Scientific) equipped with a thermostated (55 °C) reversed‐phase column (Nucleodur C18 Gravity 5 μM, 250 mm×4.6 mm) and a UV detector (205 nm). Elution was performed for 5 min with buffer A (aqueous 0.05 % trifluoroacetic acid) and then for 45 min with a linear 0 to 100 % gradient of buffer B (aqueous 0.035 % trifluoroacetic acid and 10 % CH3CN) at a flow rate of 0.8 mL.min^−1^.

Low‐resolution mass spectrometry analyses (ESI) were recorded with an ESQUIRE 3000+ ion trap mass spectrometer (Bruker/Daltonics, Wissembourg, France) and HRMS spectra were recorded with a LTQ‐Orbitrap XL mass spectrometer (Thermo Fisher Scientific).

**Strains and plasmids**: *E. coli* TOP 10 cells (Invitrogen) were used for plasmid propagation and the *E. coli* K12 strain DH1 (DSM 4235) was used for metabolic engineering and muropeptide production. The plasmids pKO3 and pEXT22 were used for *nagZ, ampD, amiD* and *ldcA* inactivation.

**Inactivation of** ***nagZ***: To knockout the *nagZ* gene from the *E. coli* K12 strain DH1 (DSM 4235), a 411 bp segment located between nucleotides 287 and 697 was deleted and replaced with the 5’‐AAGCTT‐3’ fragment. Two DNA segments flanking the deleted sequence were PCR amplified from *E. coli* K12 genomic DNA. The upstream 843 bp segment was amplified with primers 5’‐AATGGATCCGGCGCGGTAAAAACGTATTTG‐3’ and 5’‐GGTAAGCTTGTTTGCCACCCTCTTCCATTCC‐3’, and the downstream 836 bp segment was amplified with primers 5’‐CCCCCTTTTAAGCTTCCGTTTTGCGTCAGGAACTG‐3’ and 5’‐ CCCCCCCTTTTGTCGACTTGTGCGTCTGCTCTTCGTC‐3’. *BamH*I and *Hind*III restriction sites were added to the 5’ and 3’ ends of the upstream segment, respectively, and *Hind*III and *Sal*I restriction sites were added to the 5’ and 3’ ends of the downstream segment, respectively. The two amplified fragments were then simultaneously cloned into the *BamH*I and *Sal*I sites of the pKO3 suicide vector (the *Hind*III sites being used to ligate both fragments resulting in the truncated *nagZ* sequence). Before performing gene disruption, the *E. coli* K12 strain DH1 (DSM 4235) having a *recA* ^*−*^ genotype was transformed with a pEXT22 plasmid that carried a functional *recA* gene and a kanamycin resistance gene. The deletion was then carried out according to the pKO3 gene replacement protocol^[42]^. After inactivation, the pEXT22 vector was cured by growing cells without kanamycin and screening for the loss of kanamycin resistance.

**Inactivation of ampD, amiD and ldcA**: The same procedure was used to knockout the three amidases.

The *ampD* gene was inactivated in the *E. coli* ▵*nagZ* strain. A 456 bp segment located between nucleotides 60 and 515 was deleted and replaced with the 5’‐AAGCTT‐3’ fragment. The upstream 701 bp segment was PCR amplified from *E. coli* K12 genomic DNA with primers 5’‐AAAGGATCCGATCAGGAAGGCATCAGAAA‐3’ and 5’‐AAAAAGCTTTCGTAATGTGGTGAGGGAAC‐3’, and the downstream 835 bp segment was amplified with primers 5’‐GGGGTATATAAGCTTACGGTTTCGTGTGCTGGTCA‐3’ and 5’‐GGGGTTTTTGTCGACACGAGGTTTTCTTCGCCATTG‐3’.

The *amiD* gene was inactivated in the *E. coli* ▵*nagZ* ▵*ampD* strain. A 546 bp segment located between nucleotides 54 and 599 was deleted and replaced with the 5’‐AAGCTT‐3’ fragment. The upstream 722 bp segment was PCR amplified from *E. coli* K12 genomic DNA with primers 5’‐AAAGGATCCAGCAGTGTCGCCAGTTTGTC‐3’ and 5’‐AAAAAGCTTCACACCCTGCCAATAACAGAG‐3’, and the downstream 722 bp segment was amplified with primers 5’‐CCGGTTTTTAAGCTTTAACTTTTACCTTGCCGGG‐3’ and 5’‐GGGGTTTTTGTCGACCGCACGATAAAGTCTTTATTCC‐3’.

The *ldcA* gene was inactivated in the *E. coli* ▵*nagZ* ▵*ampD* ▵*amiD* strain. A 313 bp segment located between nucleotides 532 and 844 was deleted and replaced with the 5’‐AAGCTT‐3’ fragment. The upstream 762 bp segment was PCR amplified from *E. coli* K12 genomic DNA with primers 5’‐AAAGGATCCCTGGCTTATTGGCATCCAC‐3’ and 5’‐AAAAAGCTTAAGATTGCCTCCCCACAAC‐3’, and the downstream 793 bp segment was amplified with primers 5’‐CCGCAAAAAAAGCTTATGCCATTCTGAATAACACCC‐3’ and 5’‐CCCCAAAAAGTCGACGTAGTAAATAACCGCCGCC‐3’.

**Media composition**: Routine cultures of *E. coli* were performed in LB medium (1 % tryptone, 0.5 % yeast extract, and 0.5 % NaCl). High cell density cultures were carried out in minimal medium with the following composition: NH_4_H_2_PO_4_ (5 g L^−1^), KH_2_PO_4_ (5 g L^−1^), citric acid (0.5 g L^−1^), KOH (1.65 g L^−1^), NaOH (0.65 g L^−1^), trace mineral solution (7.5 mL L^−1^), glycerol (3 g L^−1^), glucose (18 g L^−1^), MgSO_4_.7H_2_O (1 g L^−1^); glucose and MgSO_4_ were autoclaved separately and added along with thiamine (4 mg L^−1^). The trace mineral solution contained nitrilotriacetic acid (13 g L^−1^), KOH (7 g L^−1^), ferric citrate (7.5 g L^−1^), MnCl_2_.4H_2_O (1.3 g L^−1^), ZnSO_4_.7H_2_O (1.2 g L^−1^), H_3_BO_3_ (0.25 g L^−1^), Na_2_MoO_4_.2H_2_O (0.15 g L^−1^), CoCl_2_.6H_2_O (0.21 g L^−1^) and CuCl_2_.2H_2_O (0.13 g L^−1^). The feeding solution contained glycerol (750 g L^−1^), MgSO_4_.7H_2_O (18 g L^−1^) and the trace mineral solution (37.5 mL L^−1^).

**Culture conditions for muropeptide production**: High cell density cultures were carried out in 3 L bioreactors (GpcBio, Perigny, France) containing 1.5 L of minimal medium. A preculture of the mutated *E. coli* strain was first grown in LB medium at 37 °C, with shaking at 180 rpm for 16 h. This preculture was used to inoculate 30 mL of LB, which was kept at 37 °C and 180 rpm for 22 h. This culture was used to inoculate the minimal medium in a bioreactor for batch fermentation. The pH of the culture medium was regulated at 6.8 by automatic addition of aqueous ammonia and the dissolved oxygen was maintained at 40 % of air saturation by manually increasing the airflow rate and automatically adjusting the stirrer speed. The initial temperature was set at 33 °C. After total consumption of the initial glucose and glycerol indicated by a sudden increase in the dissolved oxygen level, the temperature was set to 28 °C and feeding started with an initial flow rate of 9 mL h^−1^. After 5 h of cultivation, the feeding rate was lowered to 5 mL h^−1^ and kept constant until the end of the culture.

**Analytical methods**: Cell growth was monitored by measuring optical density at 600 nm. Culture aliquots (1 mL) were taken at different time points throughout fermentation. Samples were centrifuged and the culture supernatants (then referred to as extracellular fractions) were separated from the pellets. Then the pellets were suspended in water and boiled for 10 min at 100 °C to lyse the cells. After centrifugation, the new supernatants containing the intracellular species were referred to as intracellular fractions. For the *E. coli* ▵*nagZ* strain, both intra‐ and extracellular fractions were analyzed using HPAEC‐PAD and TLC (eluting system: acetonitrile:water 8 : 2 v/v). For the other strains, both intra‐ and extracellular fractions were analyzed using TLC (eluting system: butanol:ethanol:water 4 : 3 : 3 v/v).

**Purification and characterization of GlcNAc‐anhMurNAc (1)**: At the end of the culture of the *E. coli* ▵*nagZ* strain, the bacterial pellet was recovered by centrifugation (17,500 g for 30 min at 4 °C). The culture medium was discarded and the cell pellet was resuspended in distilled water (500 mL). The cells were permeabilized by autoclaving at 100 °C for 45 min. After resuspension in distilled water (500 mL), the mixture was centrifuged (17,500 g, 4 °C, 30 min), and supernatant containing **1** was recovered.

The purification was performed in three steps. First, **1** was adsorbed on charcoal:celite (1 : 1 w/w). The slurry was filtered and washed with distilled water. Disaccharide **1** was eluted with aqueous EtOH (1 : 1 v/v). Then, **1** was purified by silica gel flash chromatography (gradient from 9 : 1 to 7 : 3 acetonitrile:water) followed by SEC in 0.1 M ammonium carbonate. After freeze‐drying, 300 mg of **1** was recovered from the intracellular fraction of a 1.5 L culture of the *E. coli* ▵*nagZ* strain. ^1^H NMR (400 MHz, D_2_O): *δ* 5.41 (s, 1H, H‐1^MurNAc^), 4.68 (d+m, *J*=8.4, 2H, H‐1^GlcNAc^, H‐5^MurNAc^), 4.24 (d, *J*=7.6 Hz, 1H, H‐6^MurNAc^), 4.18 (q, *J*=6.8 Hz, 1H, H‐α^D−Lac^), 4.07 (m, 1H, H‐2^MurNAc^), 4.00 (m, 1H, H‐4^MurNAc^), 3.93 (dd, *J*=1.6 Hz, *J*=12.4 Hz, 1H, H‐6^GlcNAc^), 3.80‐3.76 (m, 3H, H‐2^GlcNAc^,H‐6’^MurNAc^,H‐6’^GlcNAc^), 3.60 (m, 1H, H‐3^GlcNAc^), 3.55 (m, 1H, H‐3^MurNAc^), 3.50 (m, 1H, H‐4^GlcNAc^), 3.48 (m, 1H, H‐5^GlcNAc^), 2.10 (s, 3H, C*H*_3_^GlcNAc^), 2.08 (s, 3H, C*H*_3_^MurNAc^), 1.36 (d, *J*=6.8 Hz, 3H, C*H*_3_^D−Lac^); ^13^C NMR (100 MHz, D_2_O): *δ* 179.9 (*C*O^D−Lac^), 175.2 (*C*O^GlcNAc^), 173.4 (*C*O^MurNAc^), 101.0 (C‐1^GlcNAc^), 99.8 (C‐1^MurNAc^), 76.03 (C‐5^GlcNAc^), 76.00 (C‐3^MurNAc^), 75.7 (C‐α^D−Lac^), 73.8 (C‐5^MurNAc^), 73.5 (C‐4^MurNAc^), 73.3 (C‐3^GlcNAc^), 69.7 (C‐4^GlcNAc^), 64.8 (C‐6^MurNAc^), 60.5 (C‐6^GlcNAc^), 55.5 (C‐2^GlcNAc^), 50.3 (C‐2^MurNAc^), 22.3 (*C*H_3_^GlcNAc^), 22.3 (*C*H_3_^MurNAc^), 18.3 (*C*H_3_^D−Lac^); HRMS (ESI‐LTQ Orbitrap) *m/z*: [M+H]^+^ calcd for C_19_H_31_O_12_N_2_: 479.18715; found: 479.18594.

## Synthesis of GlcNAc‐anhMurNAc‐D‐Ala‐D‐*iso*‐Gln‐L‐Lys‐D‐Ala‐D‐Ala (2) and GlcNAc‐anhMurNAc‐D‐Ala‐D‐*iso*‐Glu‐L‐Lys‐D‐Ala‐D‐Ala (3)

**GlcNAc‐anhMurNAc‐D‐Ala‐D‐*iso*‐Gln‐L‐Lys‐D‐Ala‐D‐Ala (2)**: To an ice‐cooled solution of **1** (9.8 mg, 20.5 μmol) and pentapeptide D‐Ala‐D‐*iso*‐Gln‐L–Lys(Fmoc)‐D‐Ala‐D‐Ala‐OMe TFA salt (21.8 mg, 1.3 eq) in DMF (2 mL) was added a solution of 1‐ethyl‐3‐[3‐(dimethylamino)propyl]carbodiimide] (EDC, 11.8 mg, 3 eq) and 1‐hydroxy‐7‐azabenzotriazole (HOAt 0.6 M in DMF, 102 μL, 3 eq), followed by *N*,*N*‐diisopropylethylamine (DIPEA, 14 μL, 4 eq). After 0.5 h at 0 °C, the mixture was stirred overnight at room temperature. Fluorenylmethoxycarbonyl (Fmoc) deprotection was carried out by adding piperidine (400 μL). After 30 min at room temperature, the solution was concentrated under reduced pressure and the residue was solubilized in H_2_O (3 mL) then centrifuged. The filtrate was purified using SEC in 0.1 M ammonium carbonate. The methyl ester was deprotected in 0.1 M ammonium carbonate at 37 °C for 48 h. Pure **2** (6 mg) was isolated with a yield of 31 % after purification by solid phase extraction on a charcoal cartridge with a 0 % to 50 % EtOH gradient in water. ^1^H NMR (400 MHz, D_2_O) *δ* 5.47 (s, 1H, H‐1^MurNAc^), 4.73 (m, 1H, H‐5^MurNAc^), 4.69 (d, *J*=8.4 Hz, 1H, H‐1^GlcNAc^), 4.36 (q, *J*=7.0 Hz, 1H, H‐α^L−Ala^), 4.30 (m, 2H, H‐α^D−Ala^, H‐α^D−*iso*−Gln^), 4.29 (m, 1H, H‐6^MurNAc^), 4.25 (m, 1H, H‐α^L−Lys^), 4.20 (q, *J*=7.0 Hz, 1H, H‐α^D−Lac^), 4.12 (q, *J*=7.0 Hz, 1H, H‐α^D−Ala−OH^), 4.02 (m, 1H, H‐2^MurNAc^), 4.00 (m, 1H, H‐4^MurNAc^), 3.92 (m, 1H, H‐6^GlcNAc^), 3.82 (m, 1H, H‐6’^MurNAc^), 3.78 (m, 1H, H‐2^GlcNAc^), 3.76 (m, 1H, H‐6’^GlcNAc^), 3.59 (m, 2H, H‐3^GlcNAc^, H‐3^MurNAc^), 3.48 (m, 1H, H‐4^GlcNAc^), 3.47 (m, 1H, H‐5^GlcNAc^), 3.01 (t, *J*=7.5 Hz, 2H, H‐ϵ^L−Lys^), 2.42 (m, 2H, H‐γ^D−*iso*−Gln^), 2.20 (m, 1H, H‐β^D−*iso*−Gln^), 2.09 (s, 3H, CH_3_^GlcNAc^), 2.07 (s, 3H, CH_3_^MurNAc^), 1.99 (m, 1H, H‐β’^D−*iso*−Gln^), 1.80 (m, 2H, H‐β^L−Lys^), 1.70 (m, 2H, H‐δ^L−Lys^), 1.44 (m, 2H, H‐γ^L−Lys^), 1.46 (d, *J*=7.5 Hz, 3H, CH_3_^L−Ala^), 1.39 (2d, 6H, *J*=7.0 Hz, CH_3_^D−Lac^, CH_3_^D−Ala^), 1.35 (d, *J*=7.0 Hz, 3H, CH_3_^D−Ala−OH^); ^13^C NMR (100 MHz, D_2_O) *δ* 179.7 (*C*O^D−Ala−OH^), 175.8, 175.7, 175.1, 175.0, 173.9, 173.5 (*C*O^D−Ala, L−Lys, D−*iso*−Gln, L−Ala, D−Lac^), 175.3 (*C*O^GlcNAc^), 173.6 (*C*O^MurNAc^), 100.5 (C‐1^GlcNAc^), 99.9 (C‐1^MurNAc^), 77.2 (C‐3^MurNAc^), 76.0 (C‐5^GlcNAc^, C‐α^D−Lac^), 74.2 (C‐4^MurNAc^), 73.6 (C‐5^MurNAc^), 73.3 (C‐3^GlcNAc^), 69.8 (C‐4^GlcNAc^), 64.8 (C‐6^MurNAc^), 60.6 (C‐6^GlcNAc^), 55.5 (C‐2^GlcNAc^), 54.0 (C‐α^L−Lys^), 52.7 (C‐α^D−*iso*−Gln^), 51.0 (C‐α^D−Ala−OH^), 49.7 (C‐α^L−Ala^), 49.5 (C‐α^D−Ala^), 48.9 (C‐2^MurNAc^), 39.1 (C‐ϵ^L−Lys^), 31.4 (C‐γ^D−*iso*−Gln^), 30.4 (C‐β^L−Lys^), 26.8 (C‐β^D−*iso*−Gln^), 26.3 (C‐δ^L−Lys^), 22.3 (*C*H_3_^GlcNAc^), 22.0 (C‐γ^L−Lys^), 21.9 (*C*H_3_^MurNAc^), 18.0 (*C*H_3_^D−Lac^), 17.4 (*C*H_3_^D−Ala−OH^), 16.6 (*C*H_3_^L−Ala^), 16.5 (*C*H_3_^D−Ala^); HRMS (ESI‐LTQ Orbitrap) *m/z*: [M+H]^+^ calcd for C_39_H_66_O_18_N_9_: 948.45203; found: 948.45179.

**GlcNAc‐anhMurNAc‐D‐Ala‐D‐*iso*‐Glu‐L–Lys‐D‐Ala‐D‐Ala (3)**: Muropeptide **3** was prepared at 42 % yield from **1** (9.4 mg, 19.7 μmol) and pentapeptide D‐Ala‐D‐*iso*‐Glu(OMe)‐L–Lys(Fmoc)‐D‐Ala‐D‐Ala‐OMe TFA salt (21.5 mg, 25.2 μmol, 1.3 eq) in DMF (1.5 mL) following the procedure described for compound **2**; ^1^H NMR (400 MHz, D_2_O): *δ* 5.43 (s, 1H, H‐1^MurNAc^), 4.69 (d, *J*=5.2 Hz, 1H, H‐5^MurNAc^), 4.65 (d, 1H, *J*=8.4 Hz, H‐1^GlcNAc^), 4.35 (q, *J*=7.2 Hz, 1H, H‐α^L−Ala^), 4.31 (q, *J*=7.2 Hz 1H, H‐α^D−Ala^), 4.26 (d, *J*=8.0 Hz, 1H, H‐6^MurNAc^), 4.19 (m, 1H, H‐α^L−Lys^), 4.17 (m, 1H, H‐α^D−Lac^), 4.14 (m, 1H, H‐α^D−*iso*−Glu^), 4.07 (q, *J*=7.6 Hz, 1H, H‐α^D−Ala−OH^), 3.98 (m, 1H, H‐2^MurNAc^), 3.97 (m, 1H, H‐4^MurNAc^), 3.88 (d, *J*=11.2 Hz, 1H, H‐6^GlcNAc^), 3.80 (m, 1H, H‐6’^MurNAc^), 3.74 (1H, H‐2^GlcNAc^), 3.73 (m, 1H, H‐6’^GlcNAc^), 3.58 (m, 1H, H‐3^MurNAc^), 3.56 (m, 1H, H‐3^GlcNAc^), 3.45 (m, 1H, H‐4^GlcNAc^), 3.43 (m, 1H, H‐5^GlcNAc^), 2.97 (t, 2H, *J*=7.5 Hz, H‐ϵ^L−Lys^), 2.29 (t, 2H, *J*_=_7.6 Hz, H‐γ^D−*iso*−Glu^), 2.12 (m, 1H, H‐β^D−*iso*−Glu^), 2.05 (s, 3H, C*H_3_* ^GlcNAc^), 2.03 (s, 3H, C*H_3_* ^MurNAc^), 1.88 (m, 1H, H‐β’^D−*iso*−Glu^), 1.77 (m, 2H, H‐β^L−Lys^), 1.66 (m, 2H, H‐δ^L−Lys^), 1.42 (m, 2H, H‐γ^L−Lys^), 1.42 (d, *J*=7.2 Hz, 3H, C*H*_3_^L−Ala^), 1.37 (d, *J*=6.8 Hz, 3H, C*H*_3_^D−Lac^), 1.34 (d, *J*=7.2 Hz, 3H, C*H*_3_^D−Ala^), 1.30 (d, *J*=7.2 Hz, 3H, C*H*_3_^D−Ala−OH^); ^13^C NMR (100 MHz, D_2_O): *δ* 179.8 (*C*O^D−Ala−OH^), 177.6 (*C*OOH^D−*iso*−Glu^), 175.7 (*C*O^D−*iso*−Gln^), 175.5 (*C*O^D−Lac^), 175.3 (*C*O^GlcNAc^), 174.1 (*C*O^L−Lys^), 173.8 (*C*O^L−Ala^), 173.58 (*C*O^D−Ala^), 173.56 (*C*O^MurNAc^), 100.5 (C‐1^GlcNAc^), 99.8 (C‐1^MurNAc^), 77.1 (C‐3^MurNAc^), 76.0 (C‐α^D−Lac^), 75.9 (C‐5^GlcNAc^), 74.4 (C‐4^MurNAc^), 73.6 (C‐5^MurNAc^), 73.3 (C‐3^GlcNAc^), 69.7 (C‐4^GlcNAc^), 64.8 (C‐6^MurNAc^), 60.5 (C‐6^GlcNAc^), 55.5 (C‐2^GlcNAc^), 54.2 (C‐α^L−Lys^), 54.2 (C‐α^D−*iso*−Glu^), 51.0 (C‐α^D−Ala−OH^), 49.6 (C‐α^L−Ala^), 49.5 (C‐α^D−Ala^), 48.8 (C‐2^MurNAc^), 39.1 (C‐ϵ^L−Lys^), 31.7 (C‐γ^D−*iso*−Glu^), 30.4 (C‐β^L−Lys^), 28.0 (C‐β^D−*iso*−Glu^), 26.2 (C‐δ^L−lys^), 22.3 (*C*H_3_^GlcNAc^), 22.0 (C‐γ^D−*iso*−Glu^), 21.9 (*C*H_3_^MurNAc^), 18.0 (*C*H_3_^D−Lac^), 17.4 (*C*H_3_^D−Ala−OH^), 17.0 (*C*H_3_^L−Ala^), 16.5 (*C*H_3_^D−Ala^); HRMS (ESI‐LTQ Orbitrap) *m/z*: [M+H]^+^ calcd for C_39_H_65_O_19_N_8_: 949.43605; found: 949.43655.

**Purification and characterization of GlcNAc‐anhMurNAc‐L‐Ala‐D‐*iso*‐Glu‐meso‐DAP (4)**: After workup and purification by adsorption on charcoal followed by SEC in 0.1 M ammonium carbonate, 456 mg of pure **4** was recovered from the intracellular fraction of a 1.5 L culture of the *E. coli* ▵*nagZ* ▵*ampD* ▵*amiD* strain. ^1^H NMR (400 MHz, D_2_O): *δ* 5.46 (s, 1H, H‐1^MurNAc^), 4.71 (d, 1H, *J*=5.6 Hz, H‐5^MurNAc^), 4.67 (d, 1H, *J*=8.4 Hz, H‐1^GlcNAc^), 4.41 (q, 1H, *J*=7.2 Hz, H‐α^L−Ala^), 4.29 (m, 1H, H‐6^MurNAc^), 4.20 (m, 1H, H‐α^D−*iso*−Glu^), 4.19 (m, 1H, H‐α^D−Lac^), 4.15 (m, 1H, H‐α^*meso*−DAP^), 4.01 (m, 1H, H‐2^MurNAc^), 3.99 (m, 1H, H‐4^MurNAc^), 3.90 (m, 1H, H‐6^GlcNAc^), 3.82 (m, 1H, H‐6’^MurNAc^), 3.77 (m, 2H, H‐2^GlcNAc^, H‐6’^GlcNAc^), 3.72 (m, 1H, H‐ϵ^*meso*−DAP^), 3.62 (m, 1H, H‐3^MurNAc^), 3.58 (m, 1H, H‐3^GlcNAc^), 3.47 (m, 1H, H‐4^GlcNAc^), 3.46 (m, 1H, H‐5^GlcNAc^), 2.30 (m, 2H, H‐γ^D−*iso*−Glu^), 2.12 (m, 1H, H‐β^D−*iso*−Glu^), 2.08 (s, 3H, C*H*_3_^GlcNAc^), 2.06 (s, 3H, C*H*_3_^MurNAc^), 1.96 (m, 1H, H‐β’^D−*iso*−Glu^), 1.94 (m, 2H, H‐δ^*meso*−DAP^), 1.84 and 1.71 (m, 2H, H‐β^*meso*−DAP^), 1.44 (m, 2H, H‐γ^*meso*−DAP^), 1.43 (d, 3H, *J*=7.2 Hz, C*H*_3_^L−Ala^), 1.40 (d, 3H, *J*=6.8 Hz, C*H*_3_^D−Lac^); ^13^C NMR (100 MHz, D_2_O): *δ* 178.9 (*C*OOH‐α^*meso*−DAP^), 177.7 (*C*O^D−*iso*−Glu^), 175.4 (*C*O^D−Lac^), 175.3 (*C*O^GlcNAc^), 174.9 (*C*O^D−*iso*−glu^), 174.7 (*C*OOH‐ϵ^meso−DAP^), 173.8 (*C*O^L−Ala^), 173.6 (*C*O^MurNAc^), 100.6 (C‐1^GlcNAc^), 99.9 (C‐1^MurNAc^), 77.0 (C‐3^MurNAc^), 76.0 (C‐α^D−Lac^), 75.9 (C‐5^GlcNAc^), 74.6 (C‐4^MurNAc^), 73.6 (C‐5^MurNAc^), 73.3 (C‐3^MurNAc^), 69.7 (C‐4^GlcNAc^), 64.8 (C‐6^MurNAc^), 60.6 (C‐6^GlcNAc^), 55.5 (C‐2^GlcNAc^), 54.9 (C‐α^*meso*−DAP^), 54.6 (C‐ϵ^*meso*−DAP^), 54.5 (C‐α^D−*iso*−Glu^), 49.4 (C‐α^L−Ala^), 48.7 (C‐2^MurNAc^), 32.0 (C‐γ^D−*iso*−Glu^), 31.2 (C‐β^*meso*−DAP^), 30.1 (C‐δ^*meso*−DAP^), 27.9 (C‐β^D−*iso*−Glu^), 22.3 (*C*H_3_^GlcNAc^), 21.9 (*C*H_3_^MurNAc^), 21.2 (C‐γ^*meso*−DAP^), 17.9 (*C*H_3_^D−Lac^), 17.2 (*C*H_3_^L−Ala^); HRMS (ESI‐LTQ Orbitrap) *m/z*: [M+H]^+^ calcd for C_34_H_55_O_19_N_6_: 851.35165; found: 851.35060.

**Purification and characterization of GlcNAc‐anhMurNAc‐L‐Ala‐D‐*iso*‐Glu‐meso‐DAP‐D‐Ala (5)**: After workup and purification by adsorption on charcoal followed by SEC in 0.1 M ammonium carbonate, 40 mg of **1** and 450 mg of a mixture of **4** and **5** (20 : 80 ratio as determined by ^1^H NMR and analytical reversed‐phase HPLC) were recovered from the intracellular fraction of a 1.5 L culture of the *E. coli* ▵*nagZ* ▵*ampD* ▵*amiD* ▵*ldcA* strain. A fraction of pure **5** was isolated using SEC for characterization. ^1^H NMR (400 MHz, D_2_O): *δ* 5.46 (s, 1H, H‐1^MurNAc^), 4.70 (d, 1H, *J*=5.6 Hz, H‐5^MurNAc^), 4.67 (d, 1H, *J*=8.4 Hz, H‐1^GlcNAc^), 4.40 (q, 1H, *J*=7.2 Hz, H‐α^L−Ala^), 4.30 (m, 1H, H‐6^MurNAc^), 4.27 (m, 1H, H‐α^*meso*−DAP^), 4.20 (m, 1H, H‐α^D−Lac^), 4.19 (m, 1H, H‐α^D−*iso*−Glu^), 4.14 (m, 1H, H‐α^D−Ala^), 4.01 (m, 1H, H‐2^MurNAc^), 3.99 (m, 1H, H‐4^MurNAc^), 3.89 (m, 1H, H‐6^GlcNAc^), 3.82 (m, 1H, H‐6’^MurNAc^), 3.77 (m, 1H, H‐6’^GlcNAc^), 3.77 (1H, H‐2^GlcNAc^), 3.72 (m, 1H, H‐ϵ^*meso*−DAP^), 3.62 (m, 1H, H‐3^MurNAc^), 3.58 (m, 1H, H‐3^GlcNAc^), 3.47 (m, 1H, H‐4^GlcNAc^), 3.46 (m, 1H, H‐5^GlcNAc^), 2.32 (t, 2H, *J*=7.6 Hz, H‐γ^D−*iso*−Glu^), 2.15 (m, 1H, H‐β^D−*iso*−Glu^), 2.08 (s, 3H, C*H*_3_^GlcNAc^), 2.06 (s, 3H, C*H*_3_^MurNAc^), 1.94 (m, 1H, H‐β’^D−*iso*−Glu^), 1.93 (m, 2H, H‐δ^*meso*−DAP^), 1.84 (m, 2H, H‐β^*meso*−DAP^), 1.49 (m, 2H, H‐γ^*meso*−DAP^), 1.44 (d, 3H, *J*=7.2 Hz, C*H*_3_^L−Ala^), 1.40 (d, 3H, *J*=7.2 Hz, C*H*_3_^L−Ala^), 1.31 (d, 3H, *J*=7.2 Hz, C*H*_3_^D−Ala^); ^13^C NMR (100 MHz, D_2_O): *δ* 179.8 (*C*O^D−Ala−OH^), 177.6 (*C*OOH^D−*iso*−Glu^), 175.6 (*C*O^D−*iso*−Glu^), 175.4 (*C*O^D−Lac^), 175.3 (*C*O^GlcNAc^), 174.7 (*C*OOH^*meso*−DAP^), 173.8 (*C*O^L−Ala^), 173.6 (*C*O^MurNAc^), 173.0 (*C*O^*meso*−DAP^), 100.6 (C‐1^GlcNAc^), 99.8 (C‐1^MurNAc^), 77.0 (C‐3^MurNAc^), 76.0 (C‐α^D−Lac^), 75.9 (C‐5^GlcNAc^), 74.6 (C‐4^MurNAc^), 73.6 (C‐5^MurNAc^), 73.3 (C‐3^GlcNAc^), 69.7 (C‐4^GlcNAc^), 64.7 (C‐6^MurNAc^), 60.5 (C‐6^GlcNAc^), 55.5 (C‐2^GlcNAc^), 54.5 (C‐ϵ^*meso*−DAP^), 54.3 (C‐α^D−*iso*−Glu^), 53.9 (C‐α^*meso*−DAP^), 51.0 (C‐α^D−Ala^), 49.5 (C‐α^L−Ala^), 48.7 (C‐2^MurNAc^), 31.7 (C‐γ^D−*iso*−Glu^), 30.8 (C‐β^*meso*−DAP^), 30.0 (C‐δ^*meso*−DAP^), 27.9 (C‐β^D−*iso*−Glu^), 22.3 (*C*H_3_^GlcNAc^), 21.9 (*C*H_3_^MurNAc^), 21.0 (C‐γ^*meso*−DAP^), 18.0 (*C*H_3_^D−Lac^), 17.4 (*C*H_3_^D−Ala^), 17.1 (*C*H_3_^L−Ala^); HRMS (ESI‐LTQ Orbitrap) *m/z*: [M+H]^+^ calcd for C_37_H_60_O_20_N_7_: 922.38876; found: 922.39023.

## Supporting Information Summary

Supporting Information: peptide synthesis protocols, HPLC chromatograms, NMR spectra of the products.

## Credit author statement

Antoine Rousseau: Conceptualization, Investigation, Writing ‐ original draft. Julie Michaud: Investigation. Stéphanie Pradeau: Investigation. Sylvie Armand: Supervision. Sylvain Cottaz: Supervision. Emeline Richard: Conceptualization, Supervision. Sébastien Fort: Conceptualization, Funding acquisition, Supervision, Writing ‐ original draft, Writing ‐ review & editing.

## Conflict of interest

The authors declare no conflict of interest.

1

## Supporting information

As a service to our authors and readers, this journal provides supporting information supplied by the authors. Such materials are peer reviewed and may be re‐organized for online delivery, but are not copy‐edited or typeset. Technical support issues arising from supporting information (other than missing files) should be addressed to the authors.

Supporting Information

## Data Availability

The data that support the findings of this study are available in the supplementary material of this article.
