# Supplementary material for: Hijacking the Peptidoglycan Recycling Pathway of Escherichia coli to Produce Muropeptides
Source: Chemistry. 2022 Dec 5;29(6):e202202991. doi: 10.1002/chem.202202991 (PMC10107939; doi:10.1002/chem.202202991)
Supplement: Supplementary file 1 — Supporting Information [file CHEM-29-0-s001.pdf]

Chemistry–A European Journal

Supporting Information

Hijacking the Peptidoglycan Recycling Pathway of *Escherichia coli* to Produce Muropeptides

Antoine Rousseau, Julie Michaud, Stéphanie Pradeau, Sylvie Armand, Sylvain Cottaz, Emeline Richard, and Sébastien Fort*

S1 : Monitoring of *E. coli* Δ*nagG* growth

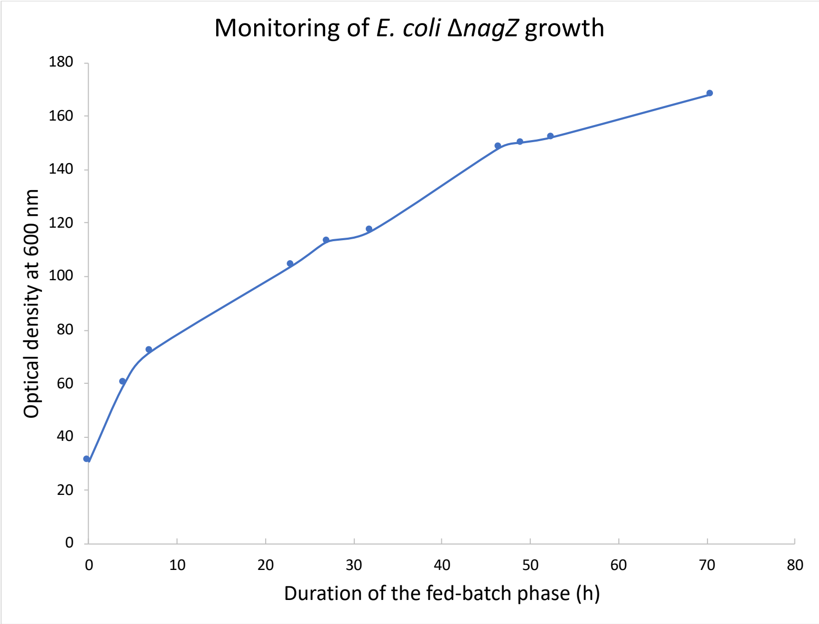

S2 : NMR spectra of **1**

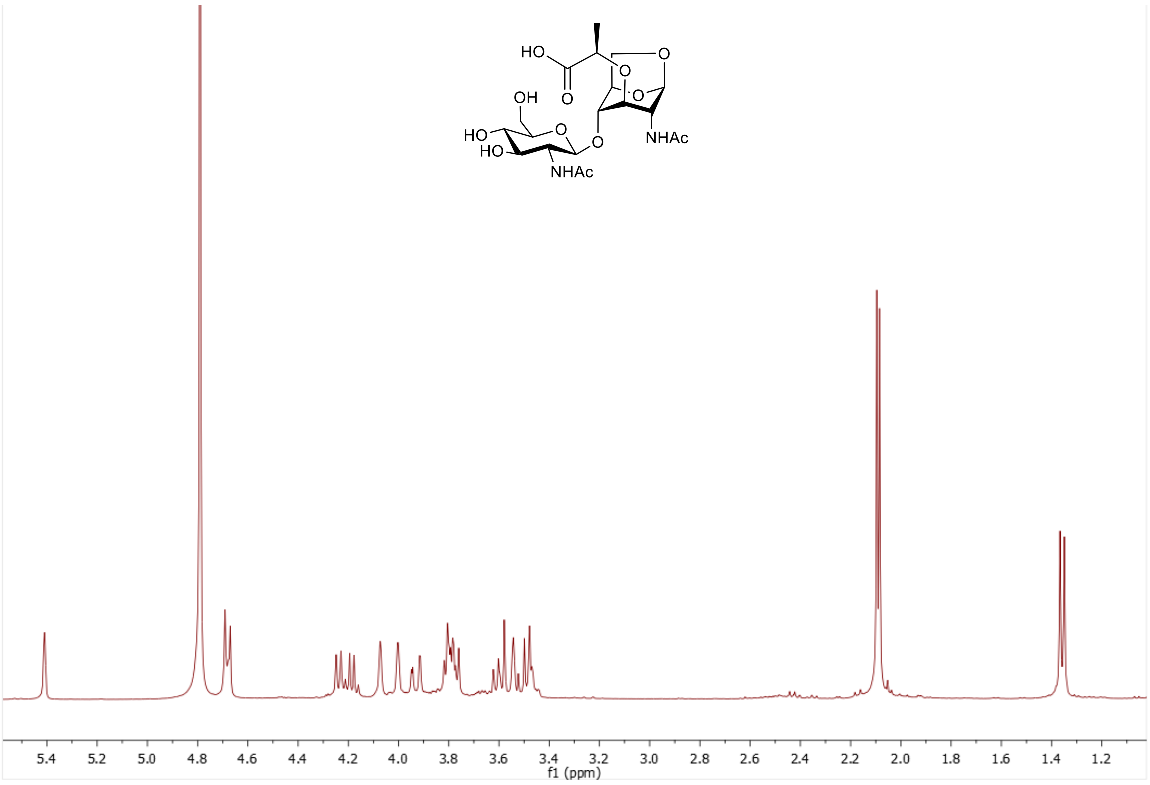

^{1}H NMR spectrum of **1** (298 K, D_2O)

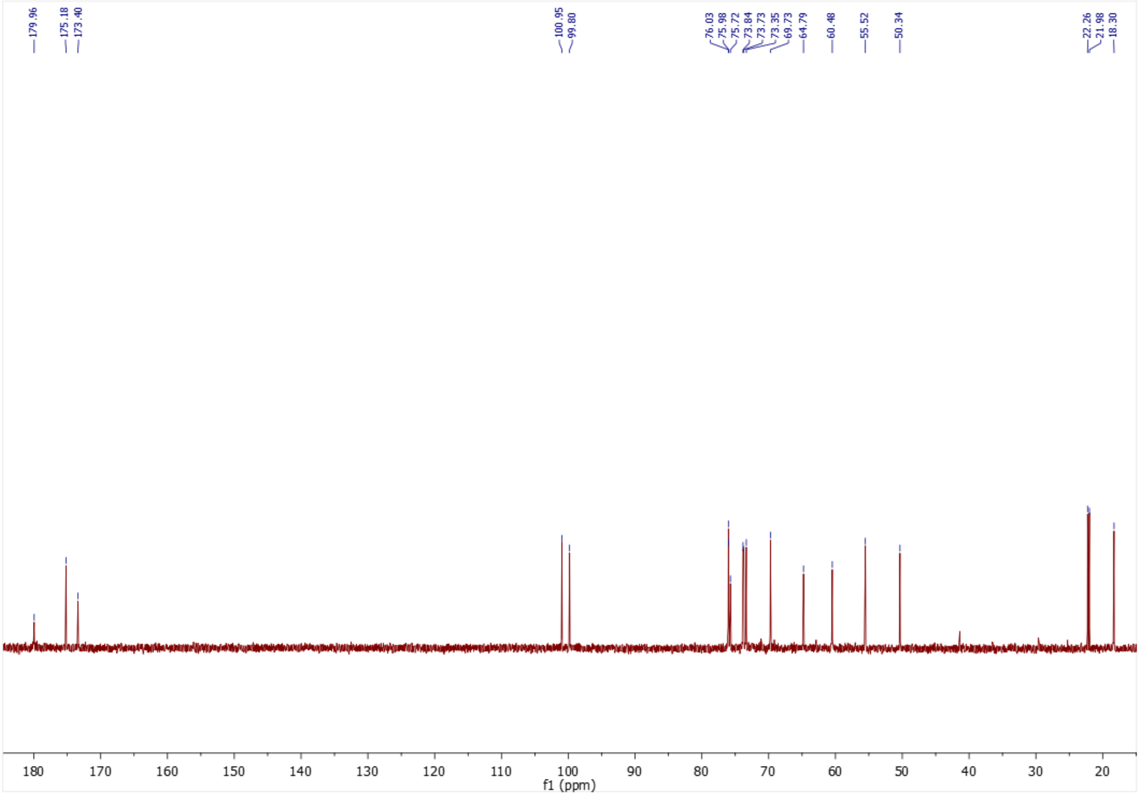

^{13}C NMR spectrum of **1** (298 K, D_2O)

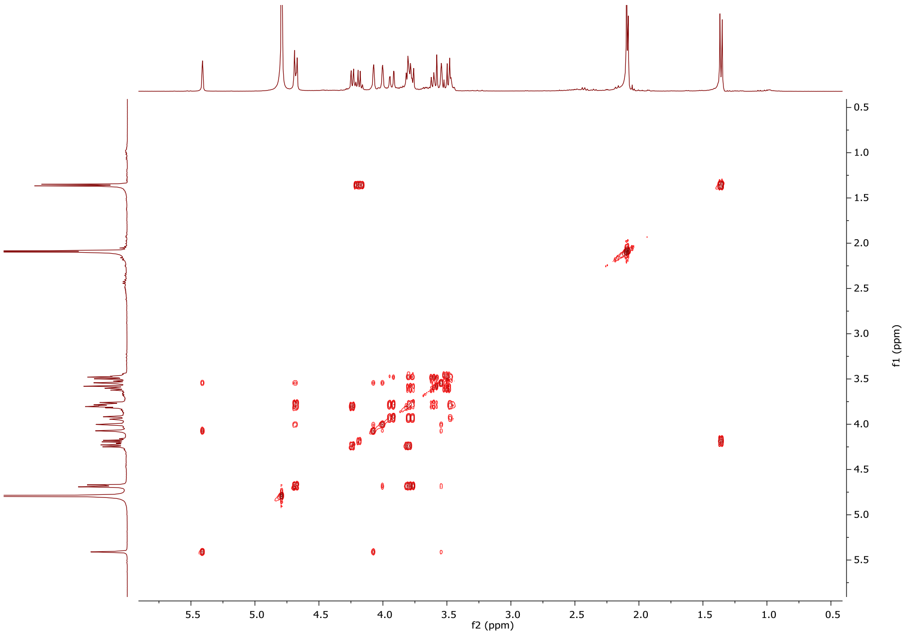

^{1}H-^{1}H COSY NMR spectrum of **1** (298 K, D_2O)

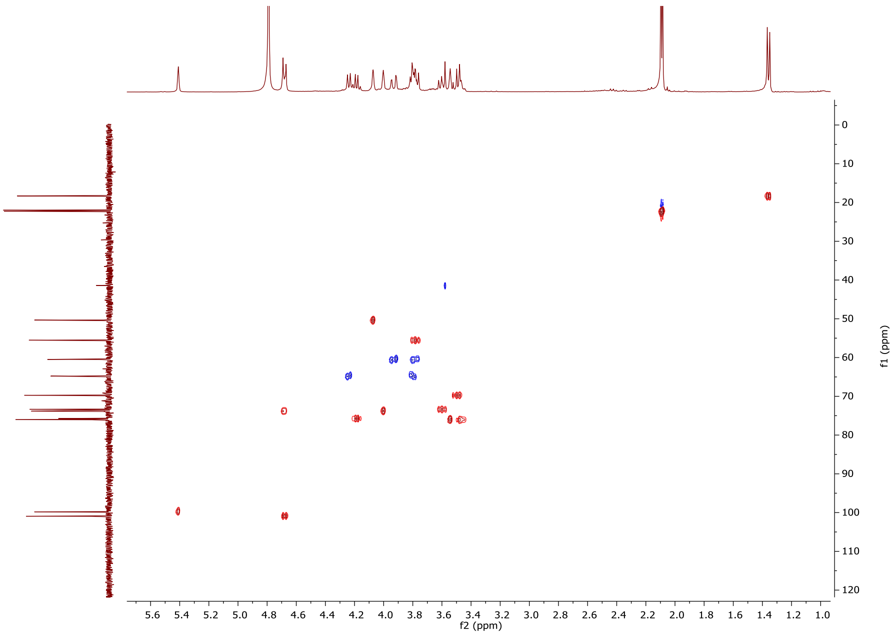

^{1}H-^{13}C HSQC NMR spectrum of **1** (298 K, D_2O). Blue signals are indicators of secondary carbons

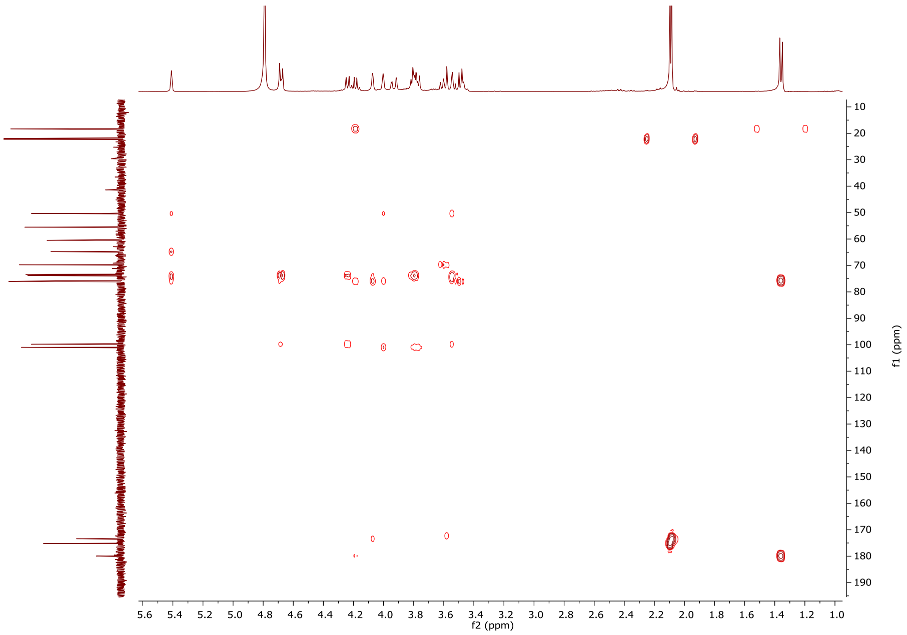

^{1}H-^{13}C HMBC NMR spectrum of **1** (298 K, D_2O)

S3 : Synthesis and characterization of peptide H-L-Ala-D-iso-Gln-L-Lys(Fmoc)-D-Ala-D-Ala(OMe) TFA salt

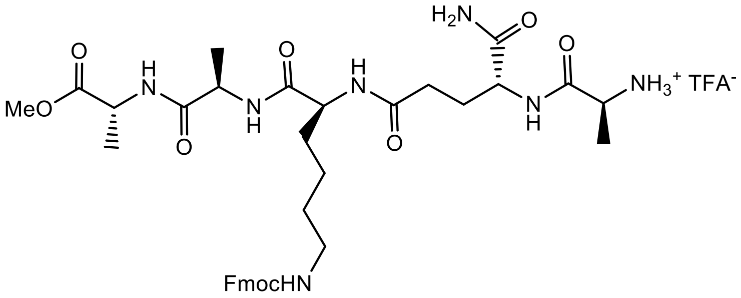

H-D-Ala-D-Ala-OH (0.510 g, 3.2 mmol) was dispersed in MeOH (35 mL) at 0°C and acetyl chloride (685 µL, 9.6 mmol) was added dropwise. The reaction was stirred for 15 min at 0°C before slowly warming up to room temperature and stirred for 22 h. After removing solvent *in vacuo* and co-evaporating with toluene, the crude was dissolved in anhydrous DMF (15 mL). DIPEA (0.743 mL, 8.0 mmol) was added. Subsequently, Boc-L-Lys(Fmoc)-OH (1.50 g, 3.2 mmol), HOAt (0.653 g, 4.8 mmol) and EDCI (0.917 g, 4.8 mmol) were added and the mixture was stirred at room temperature overnight. Water (60 mL) was poured into the reaction mixture.

Then, EtOAc (2 x 50mL) was used for the extraction and the combined organic layers were washed with water (5 x 60 mL), brine (100 mL) and dried over Na_2SO_4. The reaction mixture was concentrated and purified by flash column chromatography (DCM:EtOAc 5:5). Tripeptide Boc-L-Lys(Fmoc)-D-Ala-D-Ala(OMe) (1.34 g, 2.14 mmol) was obtained with a yield of 67% as a white solid.

Boc-L-Lys(Fmoc)-D-Ala-D-Ala(OMe) (0.580 g, 0.93 mmol) was dissolved in TFA:DCM (50:50, 6 mL) and stirred at room temperature. After 30 min, the crude was evaporated and co-evaporated with toluene (6 times) and with ether (2 times). The solid, was dissolved in anhydrous DMF (6 mL) and DIPEA (0.4 mL, 2.3 mmol) was added. Subsequently, Boc-L-Ala-D-*iso*-Gln-OH (0.294 g, 0.9 mmol), HOBt (0.189 g, 1.4 mmol) and EDCI (0.267 g, 1.4 mmol) were added and the mixture was stirred at room temperature overnight. Water (50 mL) was poured into the reaction mixture. Then EtOAc (2 x 50 mL) was used for the extraction and the combined organic layers were washed with water (5x50 mL), brine (50 mL) and dried over Na_2SO_4. The reaction mixture was filtrated on celite and rinsed with DMF. After removing solvent under reduced pressure, a white yellowish solid Boc-L-Ala-D-*iso*-Gln-L-Lys(Fmoc)-D-Ala-D-Ala(OMe) (0.746 g, 0.9 mmol) was obtained. Because of a poor solubility, the product was not purified.

^{1}H NMR (400 MHz, DMF-*d7*) : δ 7.93 (d, *J* = 7.5 Hz, 2H), 7.73 (d, *J* = 7.5 Hz, 2H), 7.44 (t, *J* = 7.5 Hz, 2H), 7.35 (t, *J* = 7.4 Hz, 2H), 4.49 – 4.08 (m, 8H), 3.66 (s, 3H), 3.11 (q, *J* = 6.6 Hz, 2H), 2.34 (t, *J* = 7.5 Hz, 2H), 2.23-2.12 (m, 1H) 1.94-1.62 (m, 5H), 1.55-1.46 (m, 2H), 1.41-1.26 (m, 18H). ESI m/z = 824.58 $[M+H]^+$

Crude Boc-L-Ala-D-*iso*-Gln-L-Lys(Fmoc)-D-Ala-D-Ala(OMe) (0.746 g) was placed in an ice bath and TFA (20 mL) was added. The mixture was stirred for 1 h, dried under reduced pressure and co-evaporated with H_2O (2 times) and ether (2 times). The obtained light yellow solid was purified by flash column chromatography with a gradient solvent system (DCM:MeOH:NH4 (9:1:0.1% 200 mL); DCM/MeOH (8:2 100 mL; 7:3 200 mL; 6:4 200 mL). TFA salt of H-L-Ala-D-*iso*-Gln-L-Lys(Fmoc)-D-Ala-D-Ala(OMe) (0.750 g, 0.89 mmol) was obtained as a white powder with a yield of 96%.

^{1}H NMR (400 MHz, MeOD): *δ* 7.83 (d, *J* = 7.5 Hz, 2H), 7.68 (d, *J* = 7.5 Hz, 2H), 7.43 (t, *J* = 7.5 Hz, 2H), 7.35 (t, *J* = 7.4 Hz, 2H), 4.52 – 4.35 (m, 5H), 4.32 – 4.19 (m, 2H), 3.80 (d, *J* = 7.0 Hz, 1H),

3.73 (s, 3H), 3.16 (t, *J* = 6.8 Hz 2H), 2.35 (t, *J* = 6.8 Hz 2H), 2.25-2.16 (m, 1H), 1.94-1.80 (m, 2H), 1.75-1.64 (m, 1H), 1.60-1.51 (m, 2H), 1.49 – 1.35 (m, 11H). ^{13}C NMR (101 MHz, DMF-*d7*): δ 173.6, 173.3, 173.2, 172.8, 172.7, 172.2, 170.1, 144.6, 141.4, 127.9, 127.3, 125.5, 120.3, 66.1, 54.1, 52.5, 51.8, 50.3, 48.6, 48.3, 40.7, 31.9, 31.6, 28.98, 28.97, 23.3, 19.2, 17.7, 16.8. ESI m/z = 724.54 $[M+H]^+$

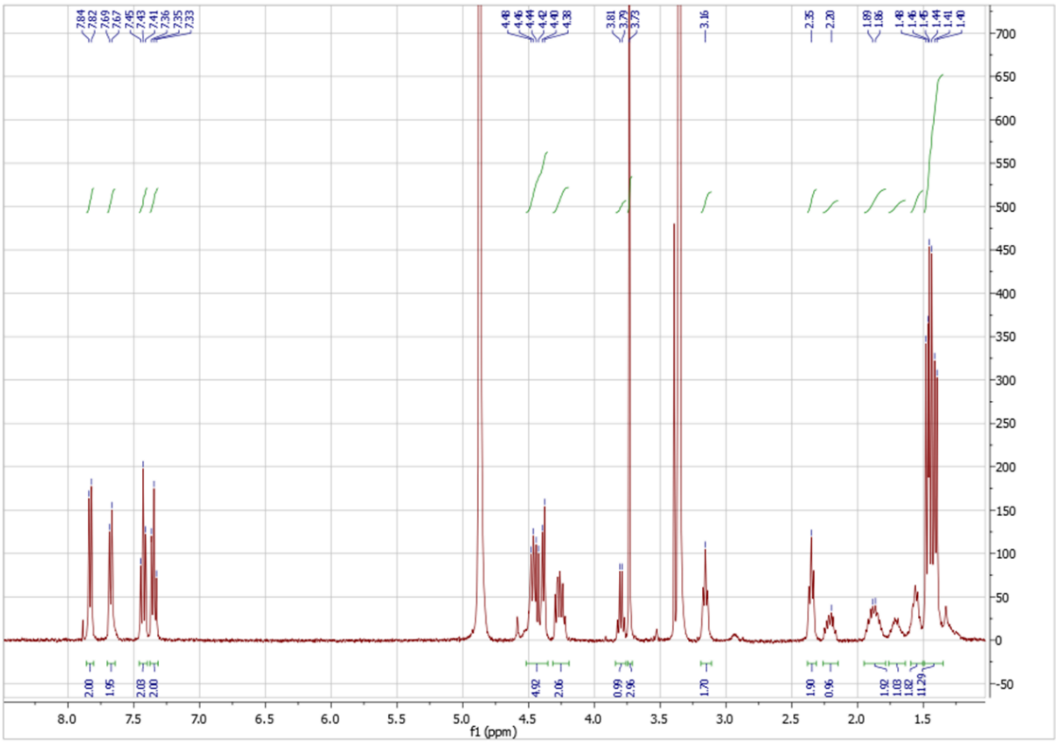

^{1}H NMR spectrum of H-Ala-D-*iso*-Gln-Lys(Fmoc)-D-Ala-D-Ala-OMe TFA Salt (298 K, MeOD)

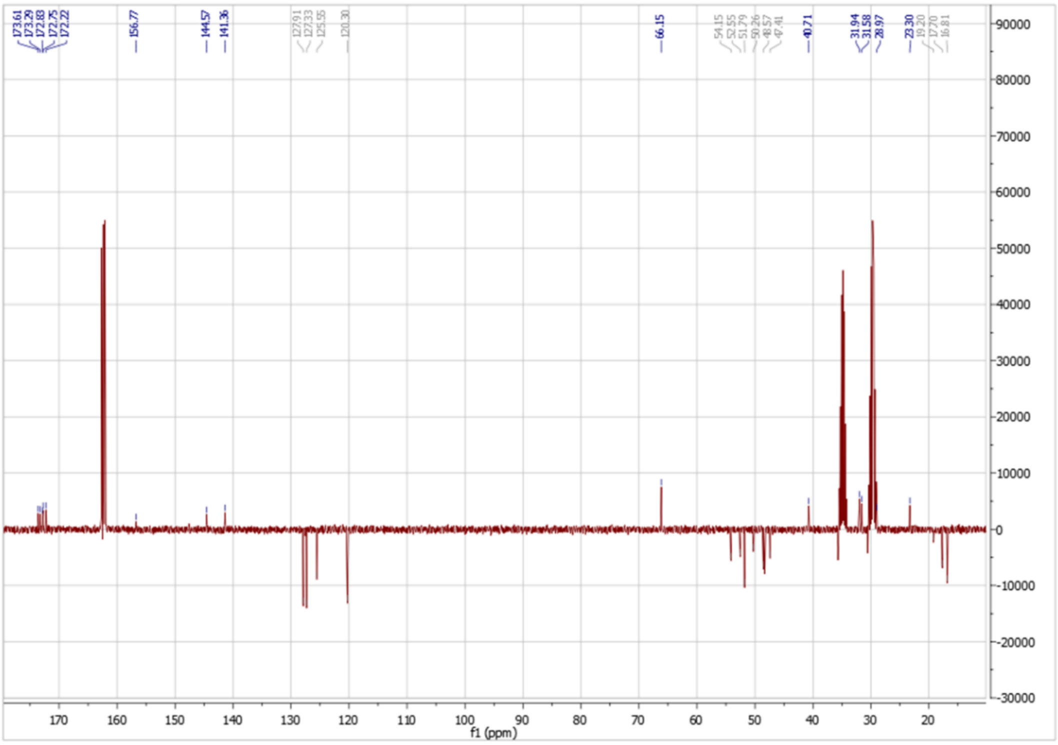

DEPTQ 135 NMR spectrum of H-L-Ala-D-*iso*-Gln-Lys(Fmoc)-D-Ala-D-Ala-OMe TFA salt (298 K, DMF-d_7)

S4 : GlcNAc-anhMurNAc-L-Ala-D-*iso*-Gln-L-Lys-D-Ala-D-Ala (**2**)

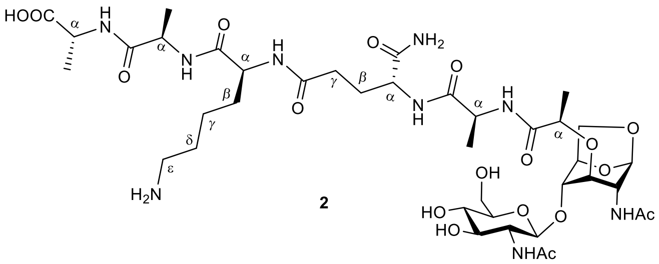

2

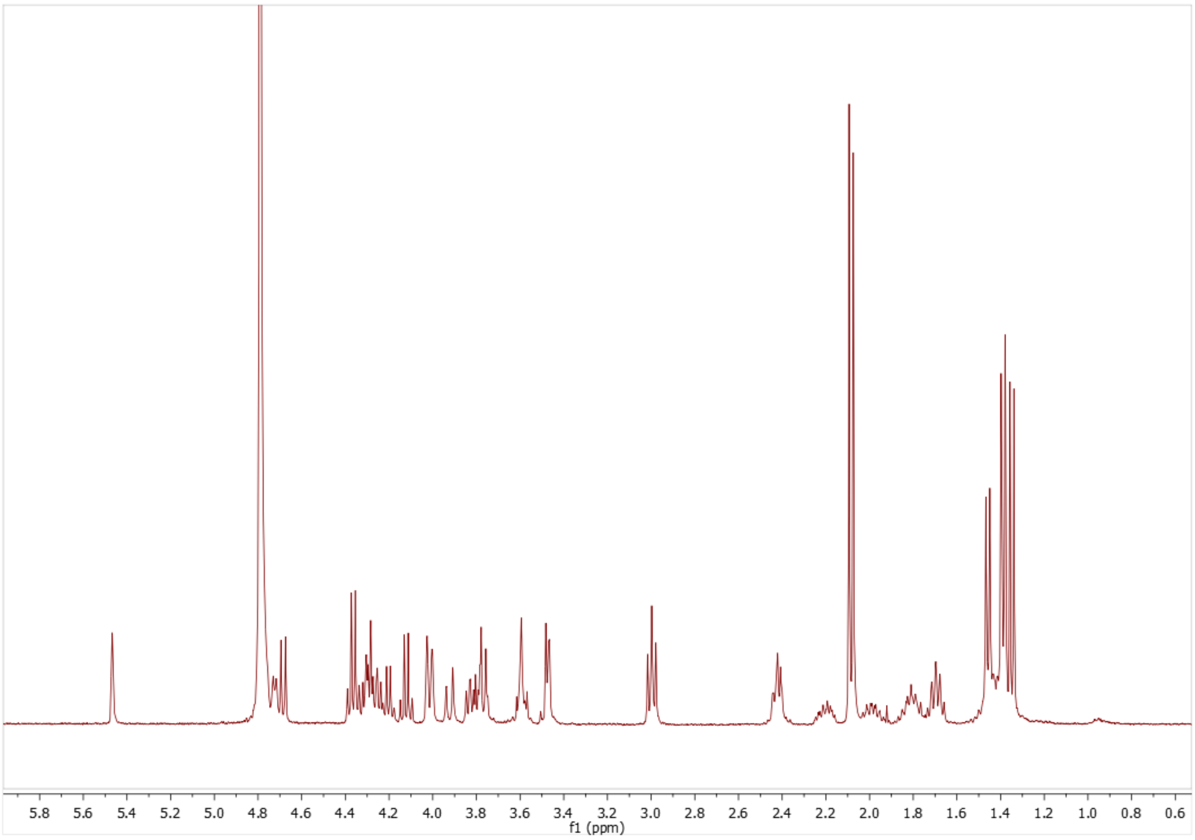

^{1}H NMR spectrum of **2** (298 K, D_2O)

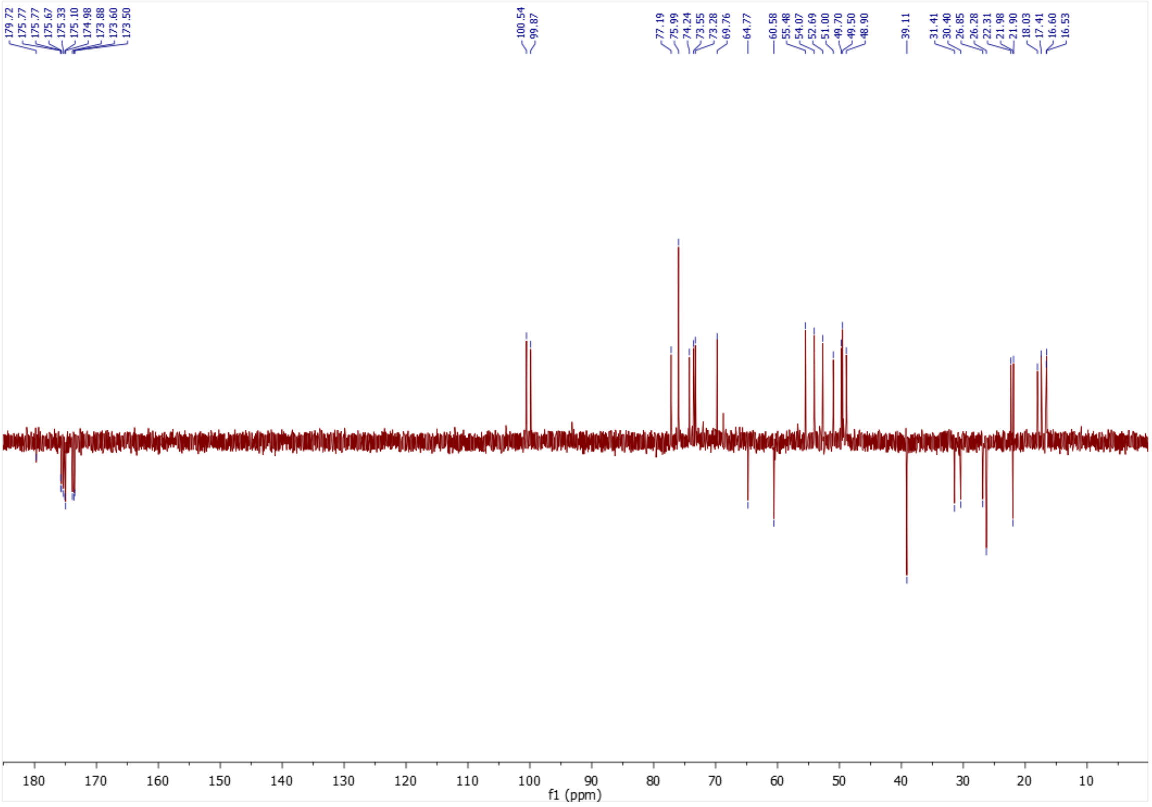

^{13}C DEPTQ 135 NMR spectrum of **2** (298 K, D_2O)

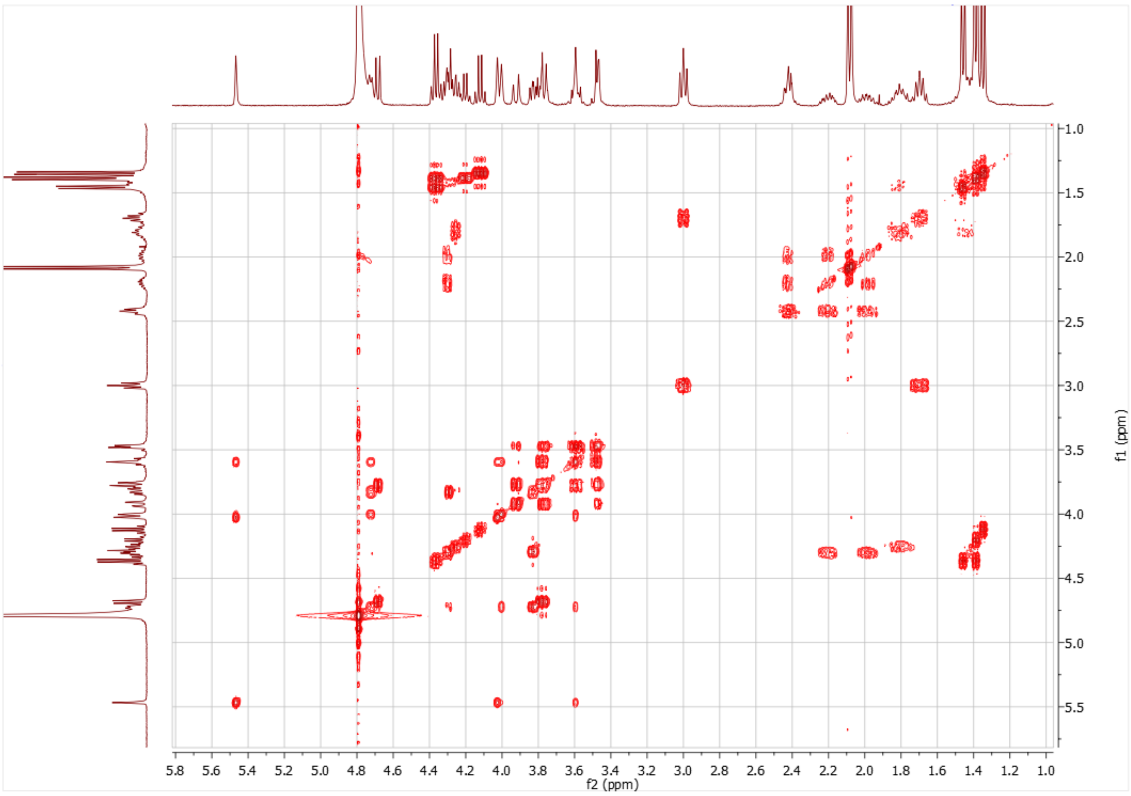

^{1}H-^{1}H COSY NMR spectrum of **2** (298 K, D_2O)

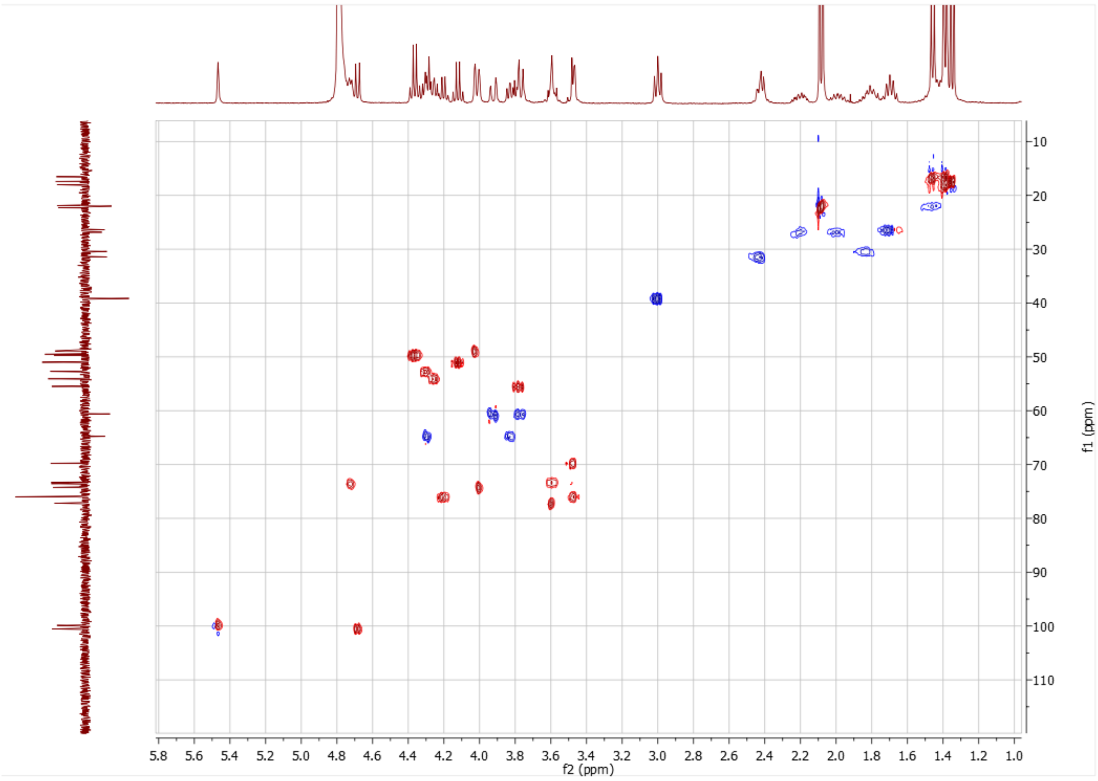

^{1}H-^{13}C HSQC NMR spectrum of **2** (298 K, D_2O). Blue signals are indicators of secondary carbons

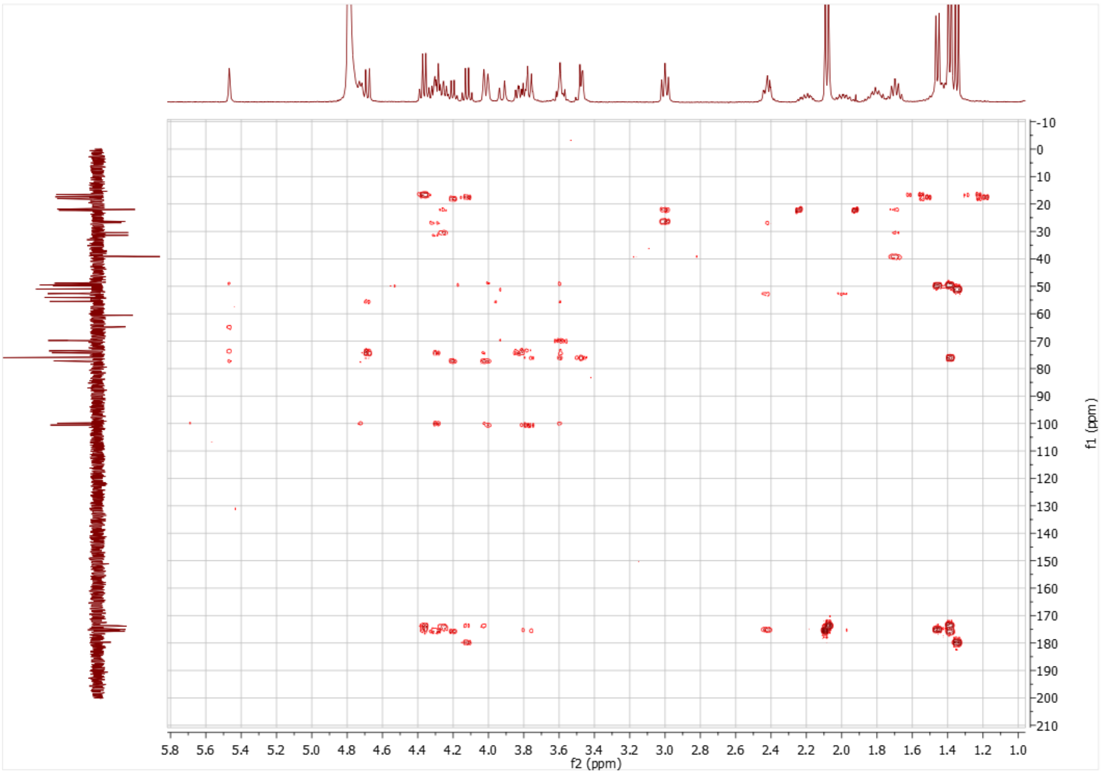

^{1}H-^{13}C HMBC NMR spectrum of **2** (298 K, D_2O)

S5 : GlcNAc-anhMurNAc-L-Ala-D-*iso*-Glu-L-Lys-D-Ala-D-Ala (**3**)

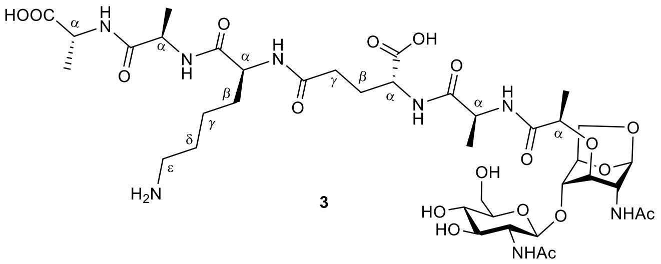

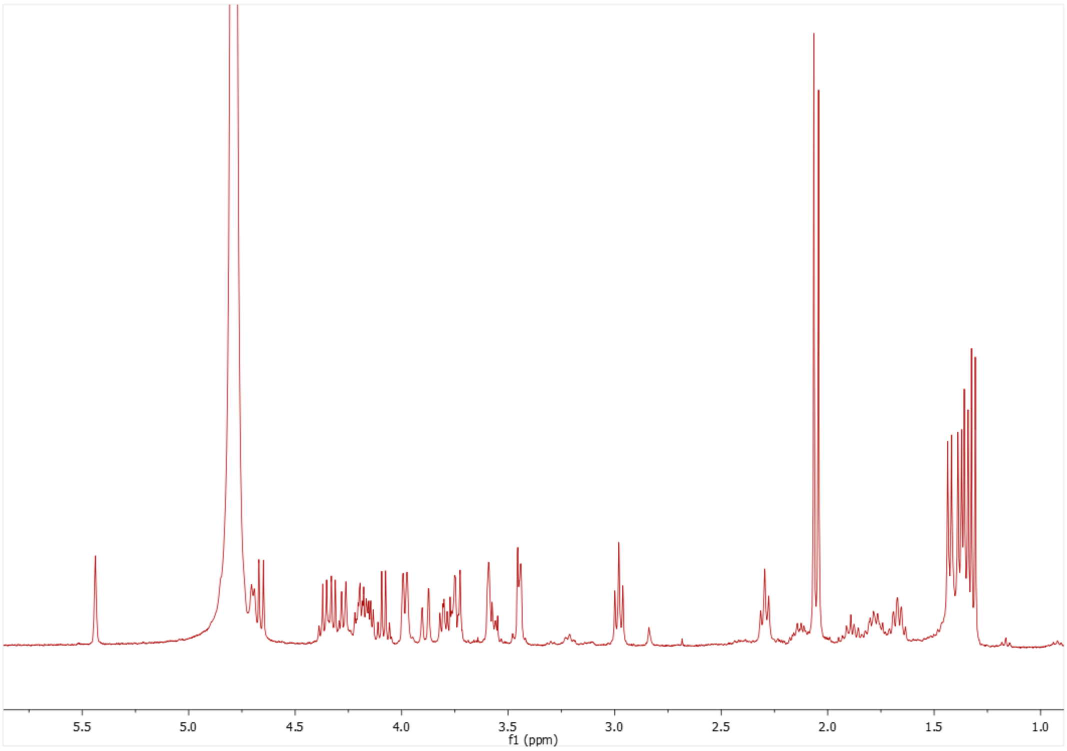

^{1}H NMR spectrum of **3** (298 K, D_2O)

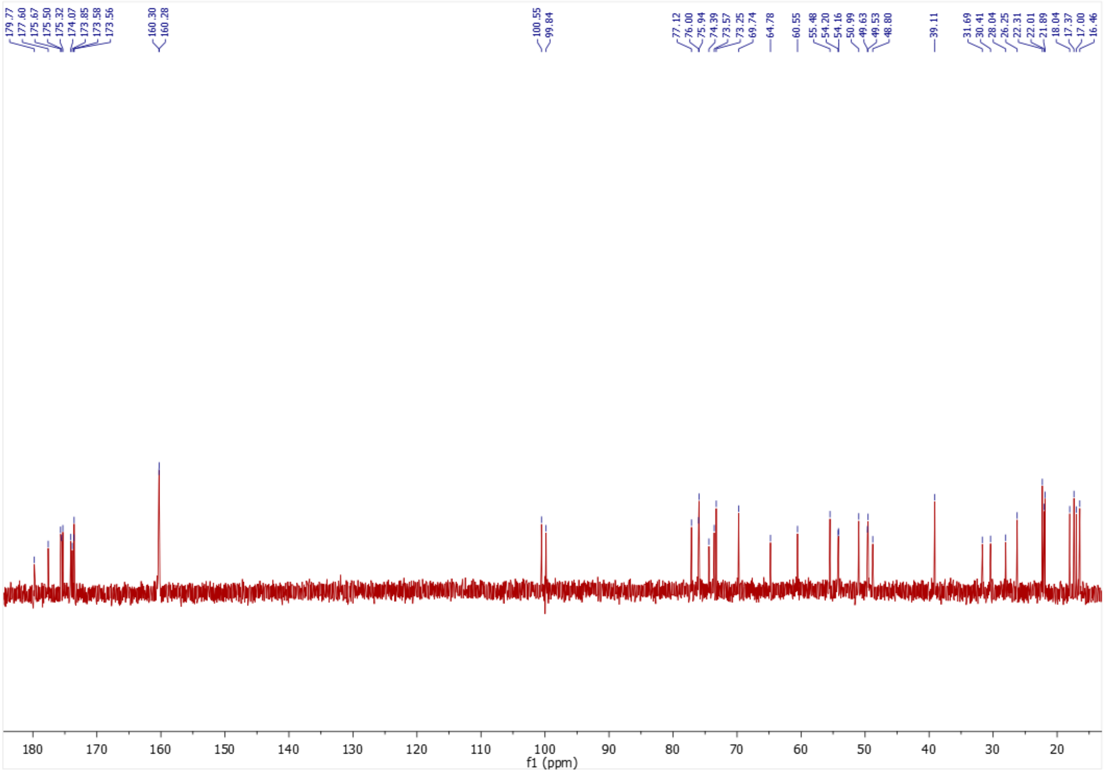

^{13}C NMR spectrum of **3** (298 K, D_2O)

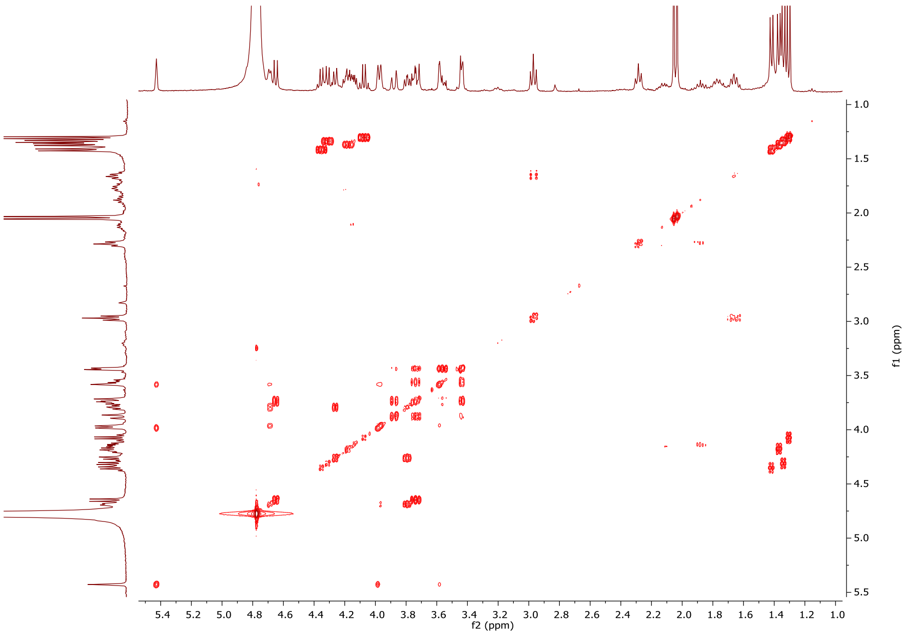

^{1}H-^{1}H COSY NMR spectrum of **3** (298 K, D_2O)

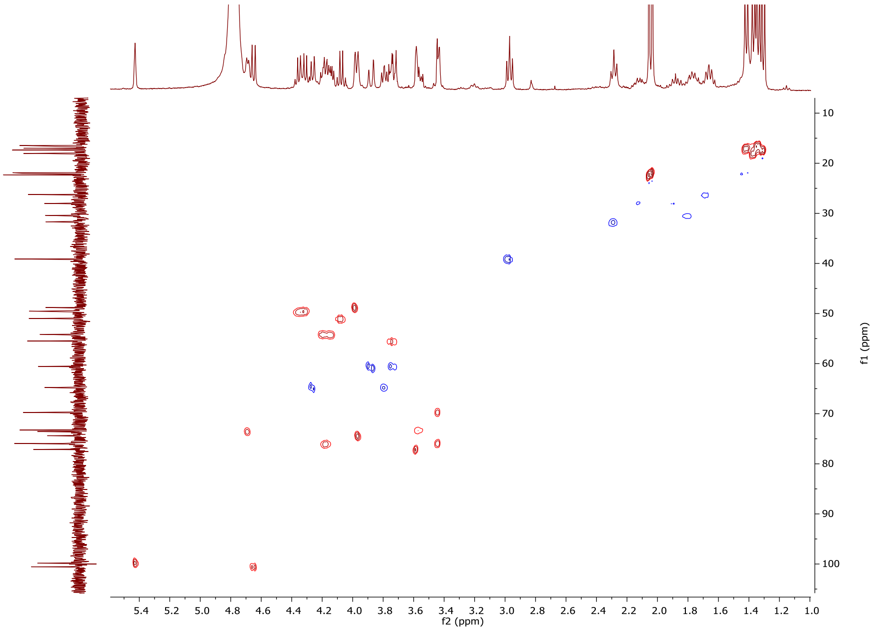

^{1}H-^{13}C HSQC NMR spectrum of **3** (298 K, D_2O). Blue signals are indicators of secondary carbons

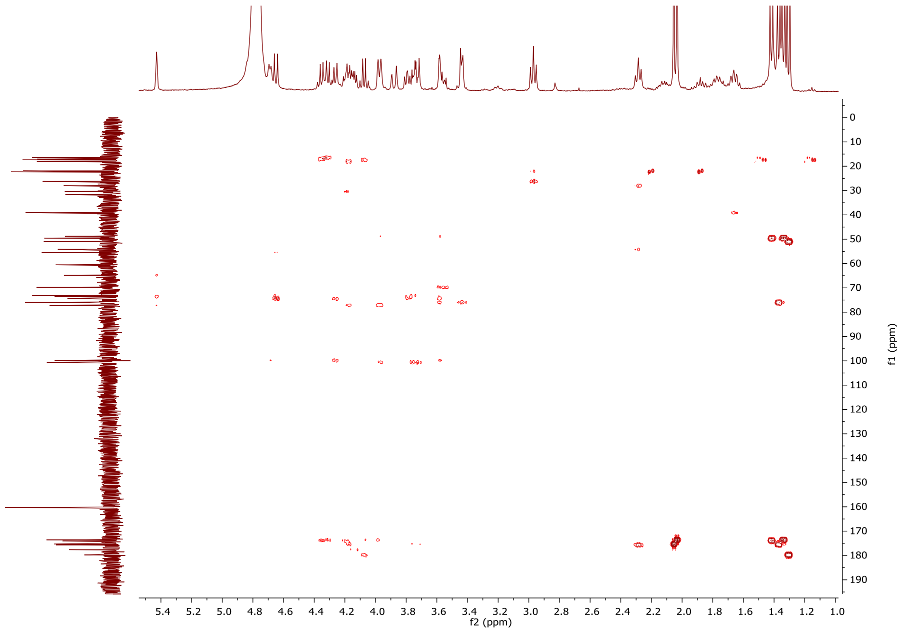

^{1}H-^{13}C HMBC NMR spectrum of **3** (298 K, D_2O)

S6: GlcNAc-anhMurNAc-L-Ala-D-*iso*-Glu-meso-DAP (**4**)

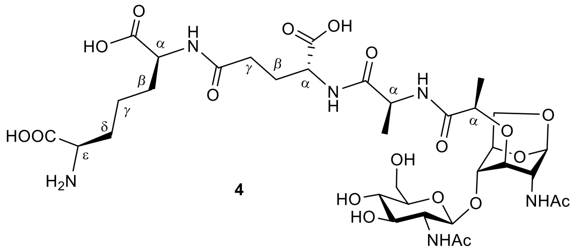

4

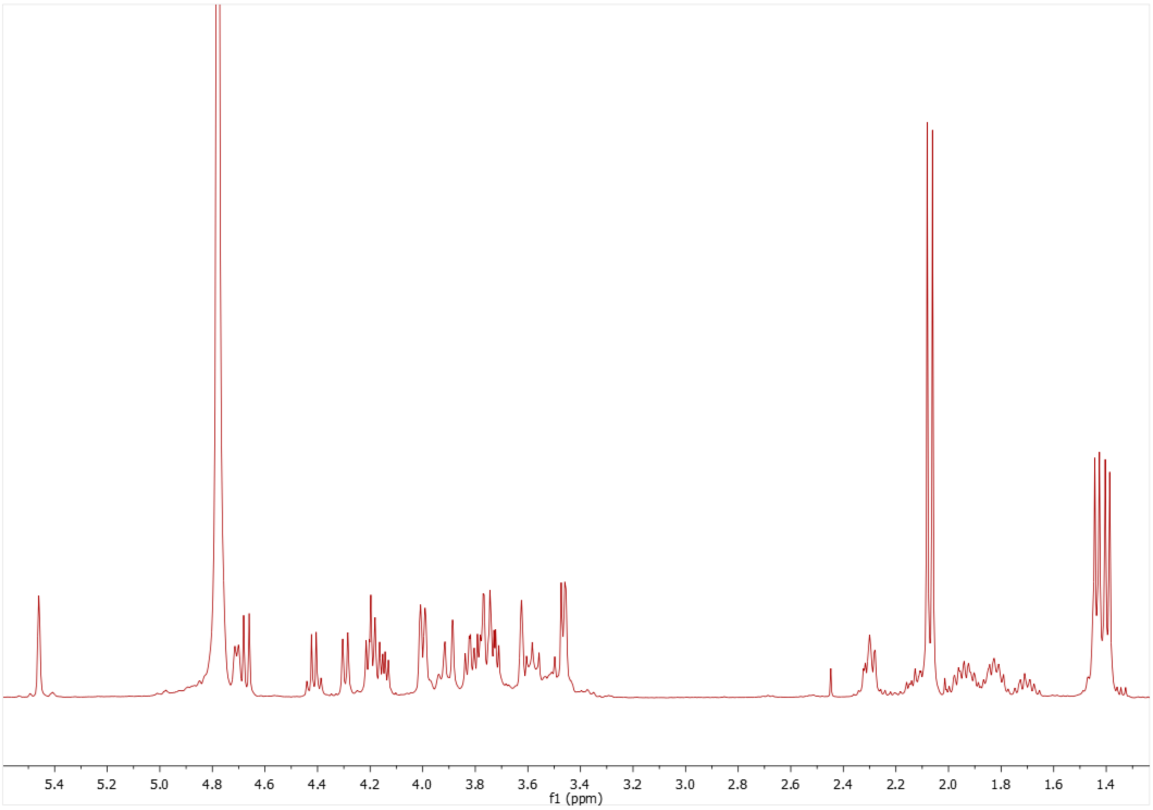

^{1}H NMR spectrum of **4** (298 K, D_2O)

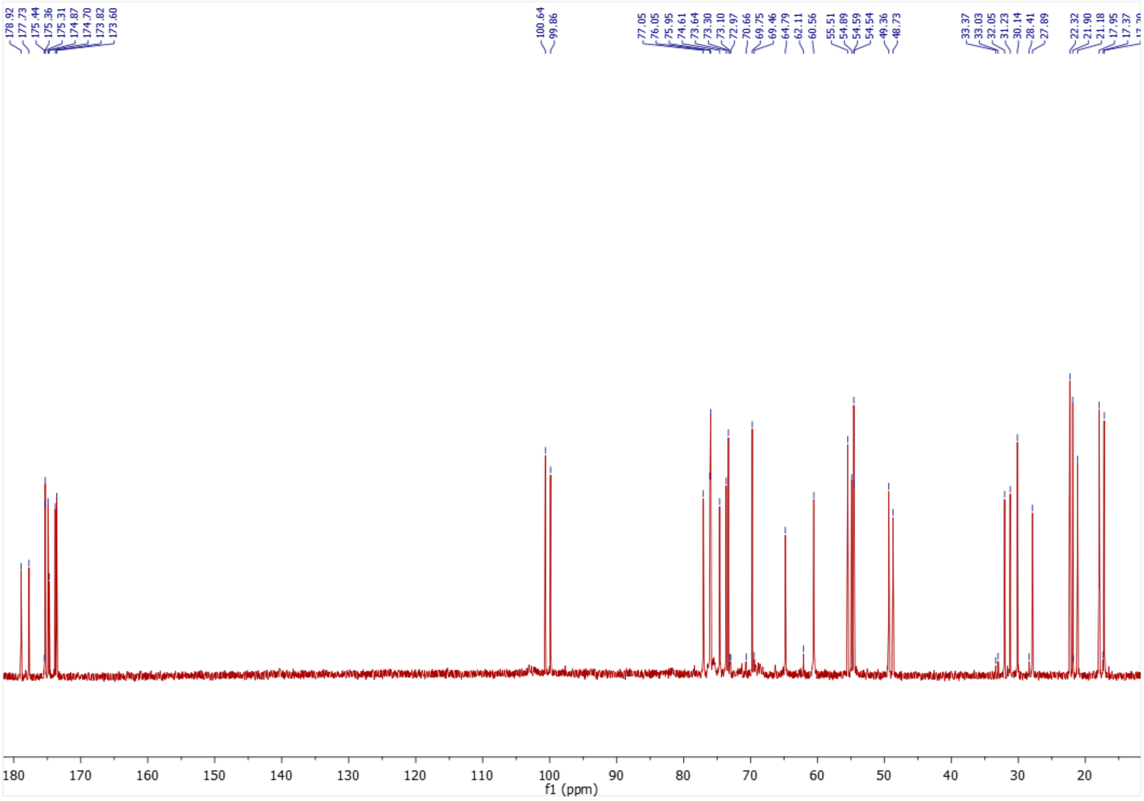

^{13}C NMR spectrum of **4** (298 K, D_2O)

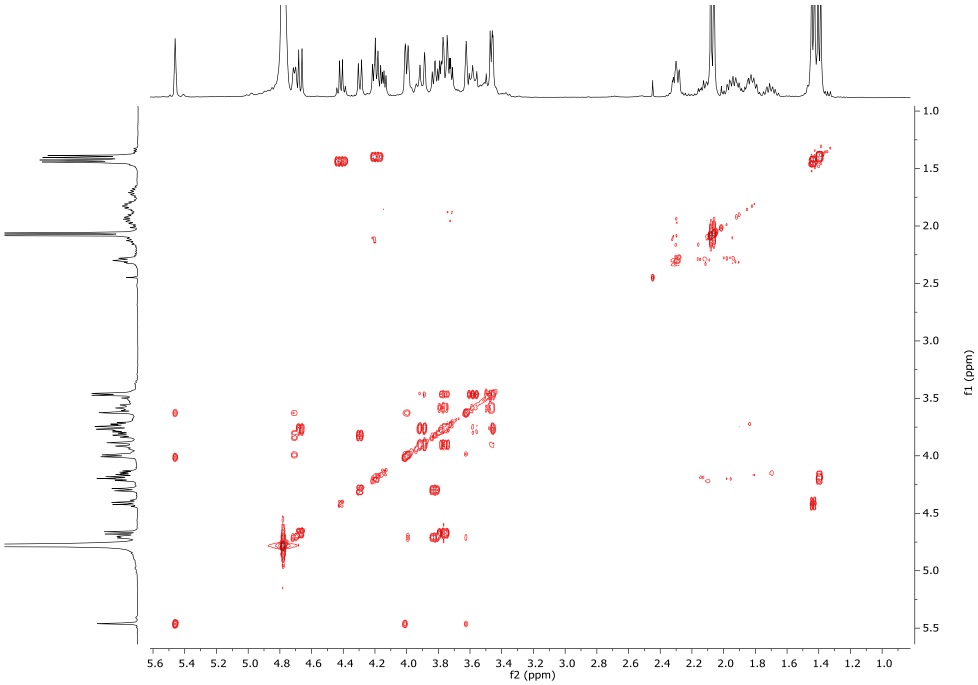

^{1}H-^{1}H COSY NMR spectrum of **4** (298 K, D_2O)

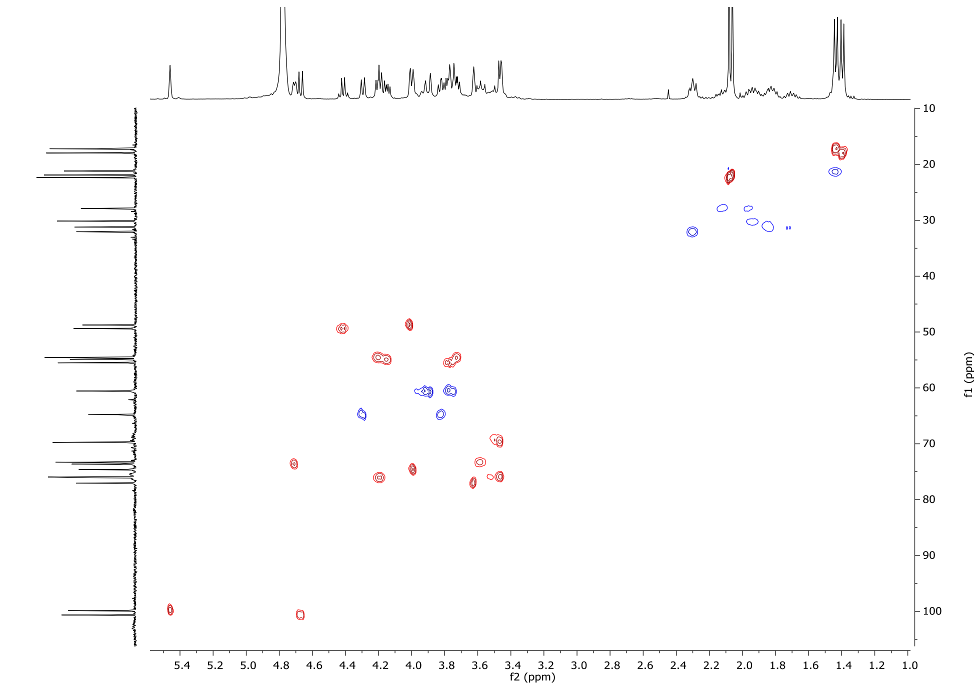

^{1}H-^{13}C HSQC NMR spectrum of **4** in D_2O. Blue signals are indicators of secondary carbons

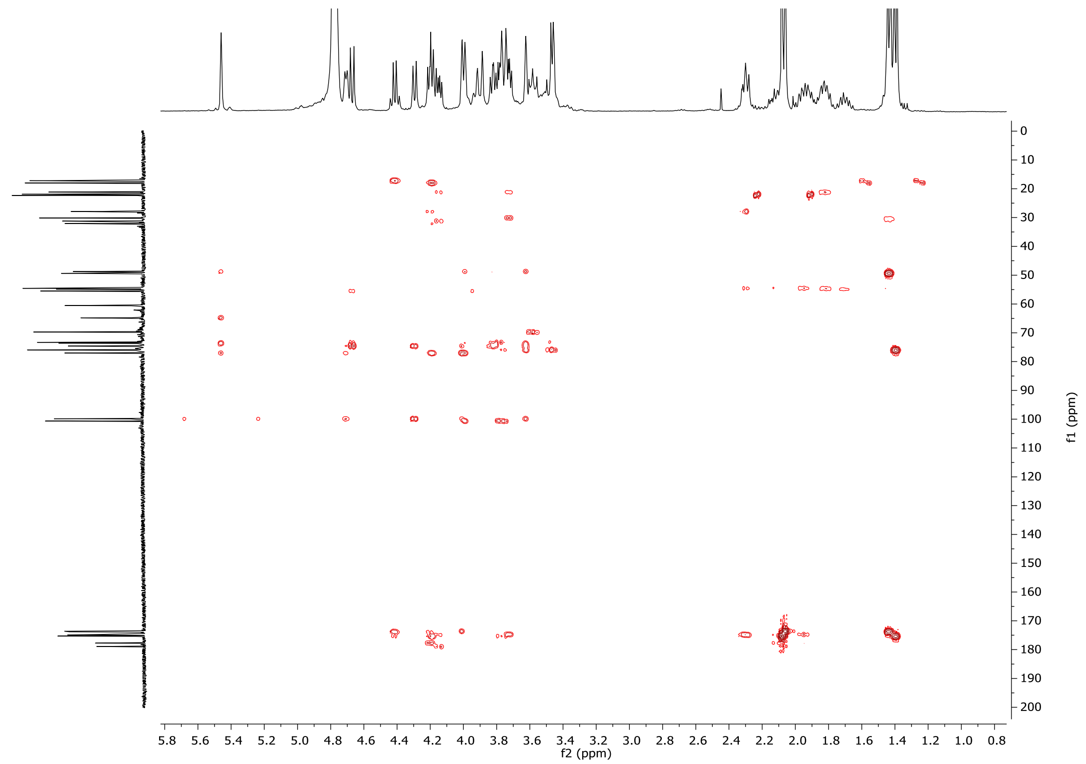

^{1}H-^{13}C HMBC NMR spectrum of **4** (298 K, D_2O)

S7: GlcNAc-anhMurNAc-L-Ala-D-*iso*-Glu-meso-DAP-D-Ala (**5**)

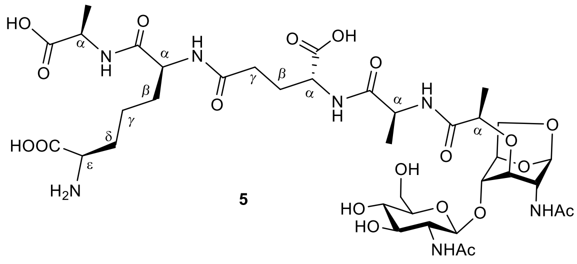

5

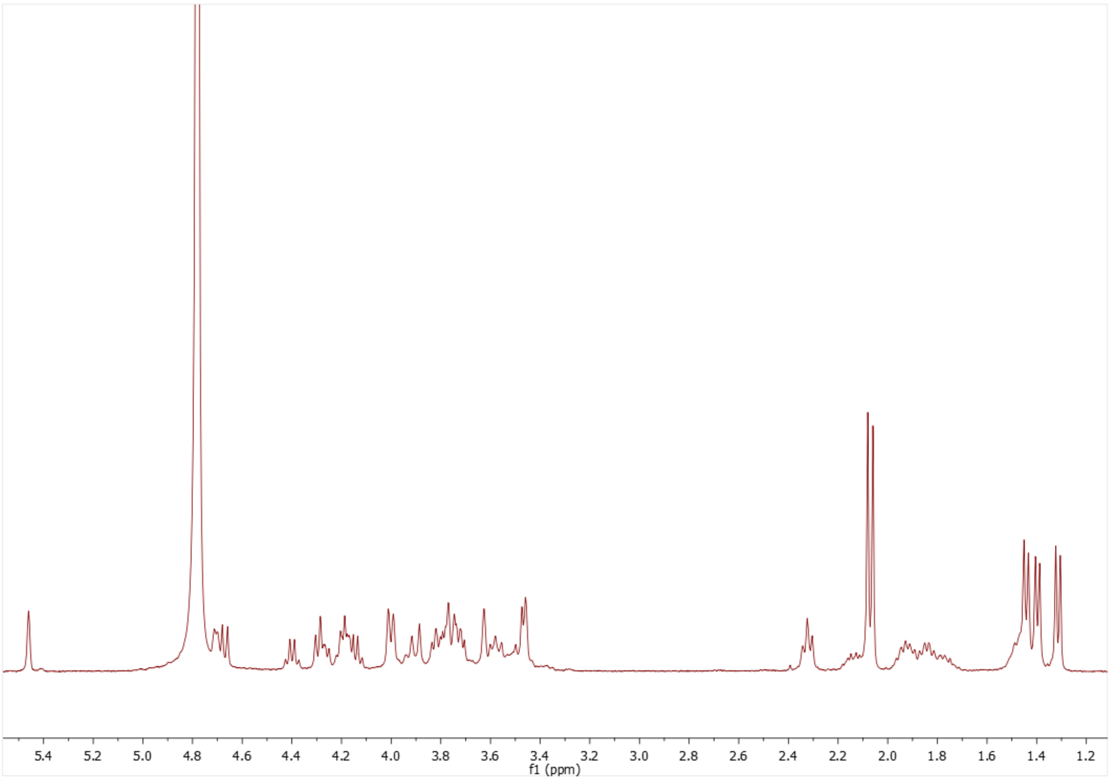

^{1}H NMR spectrum of **5** (298 K, D_2O)

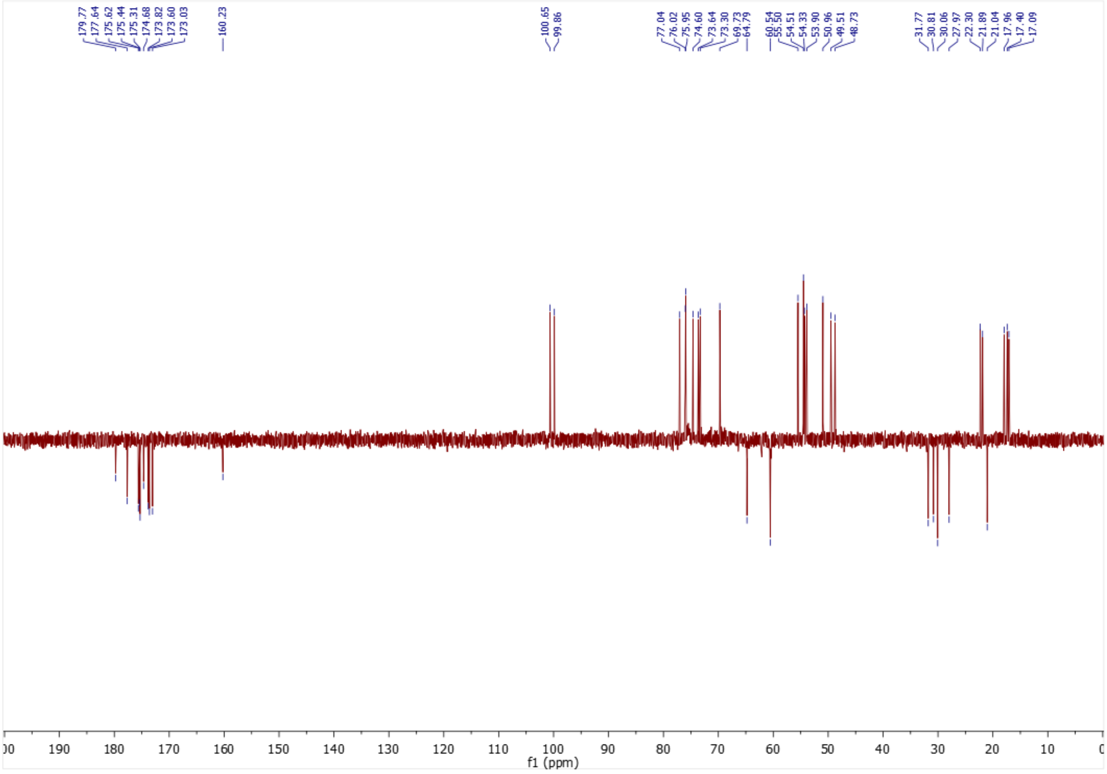

^{13}C DEPTQ 135 NMR spectrum of **5** (298 K, D_2O)

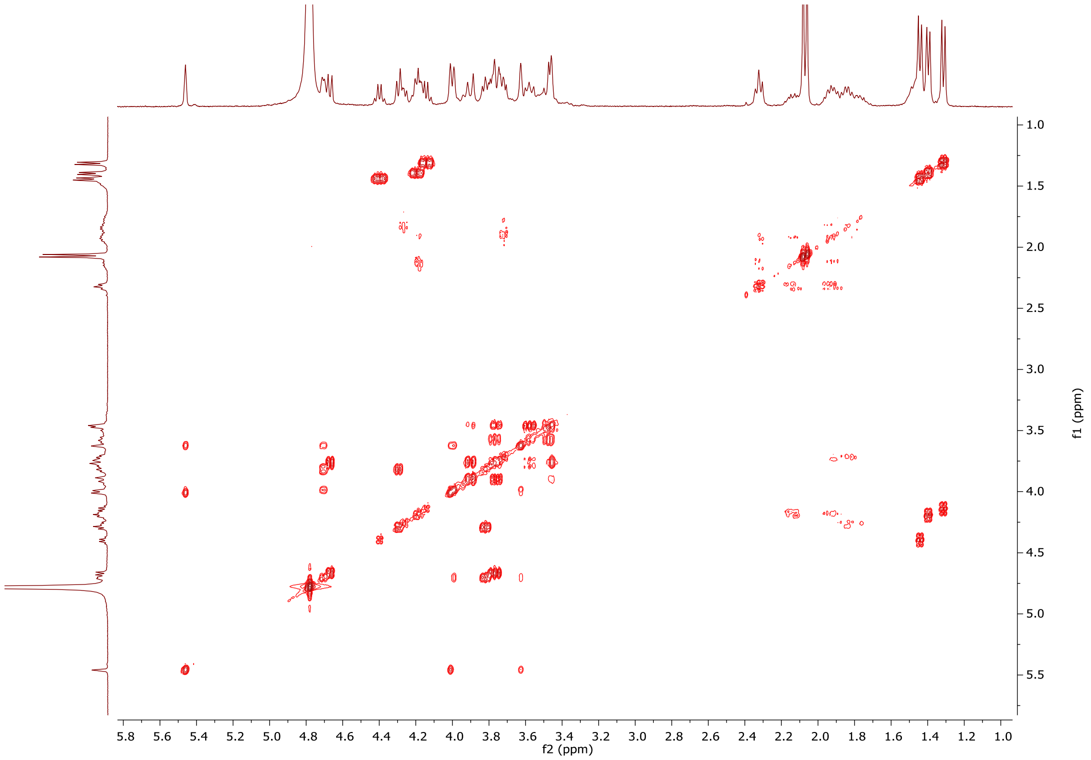

^1H-^1H COSY NMR spectrum of **5** in D_2O

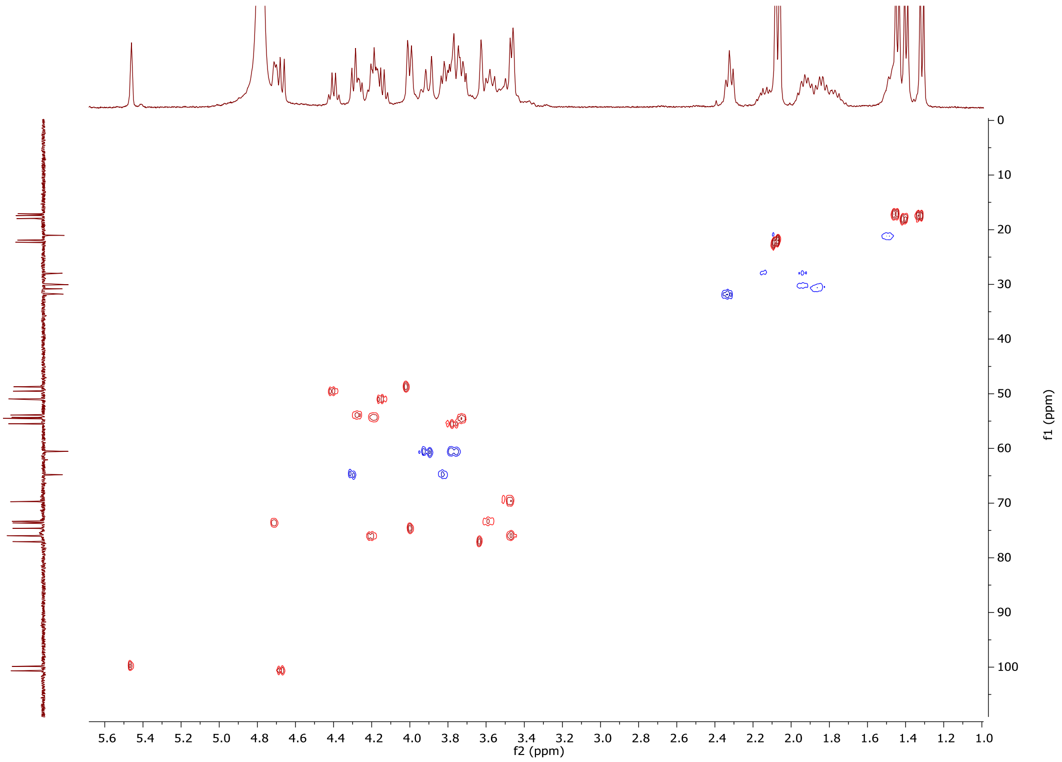

^1H-^{13}C HSQC NMR spectrum of **5** (298 K, D_2O). Blue signals are indicators of secondary carbons

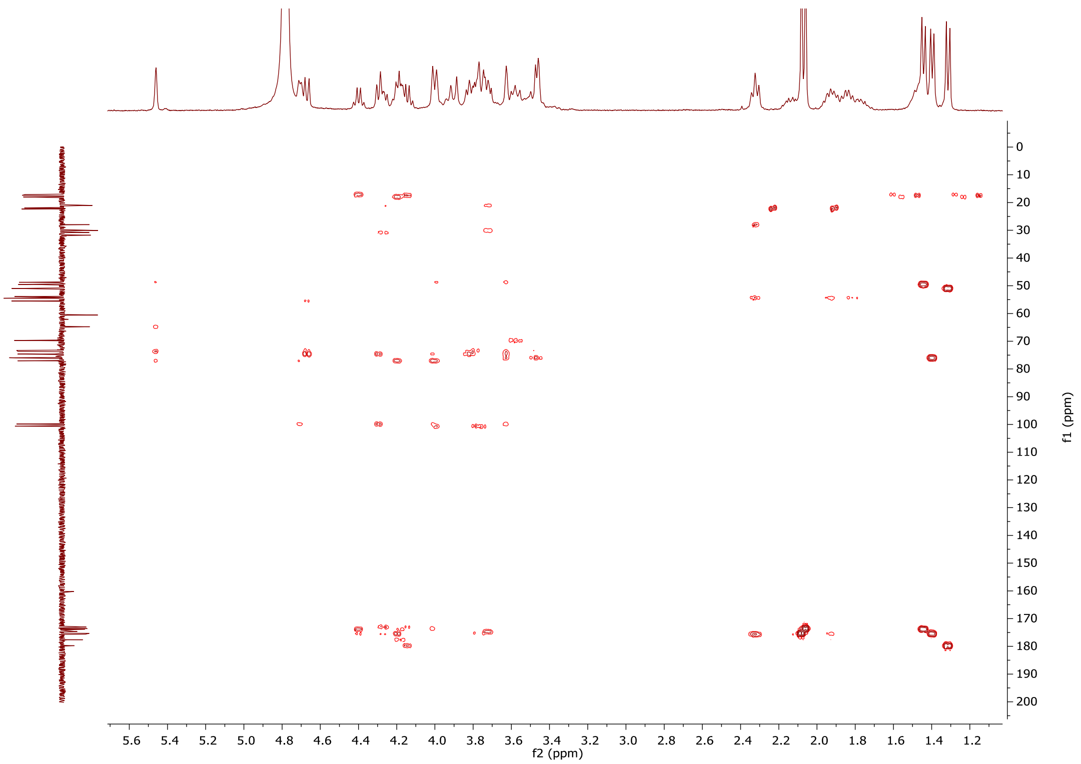

^1H-^{13}C HMBC NMR spectrum of **5** (298 K, D_2O)

S8 : Separation of tri- and tetrapeptide GlcNAc-anhMurNAc **4** and **5** by HPLC

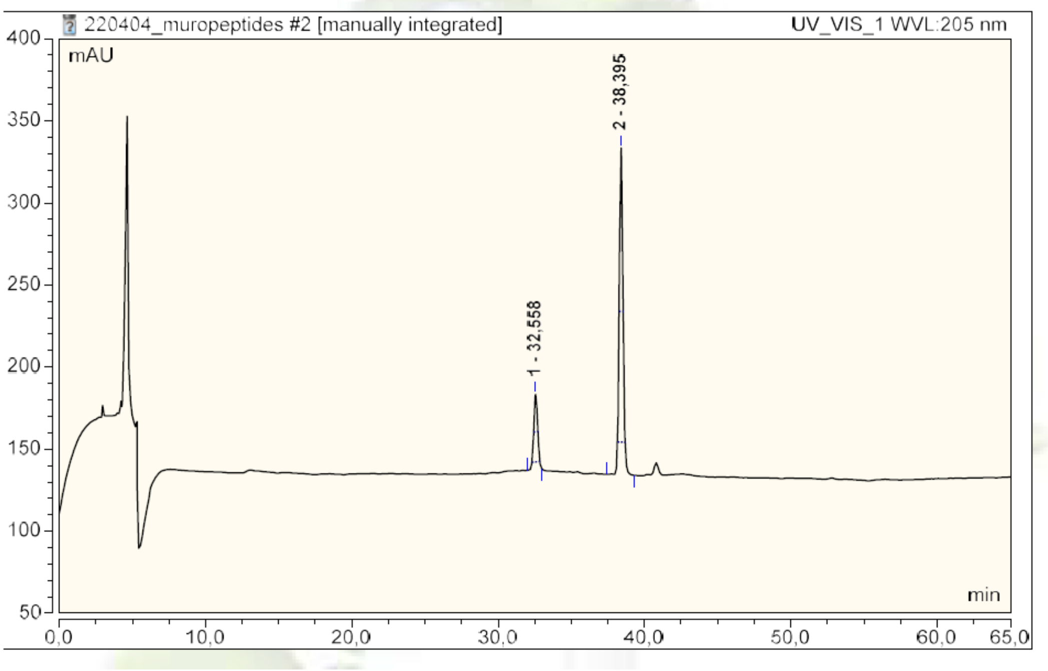

Chromatogram of the 20/80 mixture of tripeptide GlcNAc-anhMurNAc **4** and tetrapeptide GlcNAc-anhMurNAc **5** separated by reversed-phase HPLC (Nucleodur C18 Gravity 5 µM, 250

mm x 4.6 mm). Peak 1 at 32 min corresponds to tripeptide GlcNAc-anhMurNAc **4** and peak 2 at 38 min corresponds to tetrapeptide GlcNAc-anhMurNAc **5**
